# Multi-Omics Approaches to Improve Clubroot Resistance in Brassica with a Special Focus on *Brassica oleracea* L.

**DOI:** 10.3390/ijms23169280

**Published:** 2022-08-17

**Authors:** Ranjan K. Shaw, Yusen Shen, Huifang Yu, Xiaoguang Sheng, Jiansheng Wang, Honghui Gu

**Affiliations:** Institute of Vegetables, Zhejiang Academy of Agricultural Sciences, Hangzhou 310021, China

**Keywords:** *Brassica oleracea*, *Plasmodiophora brassicae*, omics, genomics, epigenomics, transcriptomics, ncRNAomics, proteomics, metabolomics

## Abstract

*Brassica oleracea* is an agronomically important species of the *Brassicaceae* family, including several nutrient-rich vegetables grown and consumed across the continents. But its sustainability is heavily constrained by a range of destructive pathogens, among which, clubroot disease, caused by a biotrophic protist *Plasmodiophora brassicae*, has caused significant yield and economic losses worldwide, thereby threatening global food security. To counter the pathogen attack, it demands a better understanding of the complex phenomenon of Brassica-*P. brassicae* pathosystem at the physiological, biochemical, molecular, and cellular levels. In recent years, multiple omics technologies with high-throughput techniques have emerged as successful in elucidating the responses to biotic and abiotic stresses. In *Brassica* spp., omics technologies such as genomics, transcriptomics, ncRNAomics, proteomics, and metabolomics are well documented, allowing us to gain insights into the dynamic changes that transpired during host-pathogen interactions at a deeper level. So, it is critical that we must review the recent advances in omics approaches and discuss how the current knowledge in multi-omics technologies has been able to breed high-quality clubroot-resistant *B. oleracea*. This review highlights the recent advances made in utilizing various omics approaches to understand the host resistance mechanisms adopted by Brassica crops in response to the *P. brassicae* attack. Finally, we have discussed the bottlenecks and the way forward to overcome the persisting knowledge gaps in delivering solutions to breed clubroot-resistant Brassica crops in a holistic, targeted, and precise way.

## 1. Introduction

*Brassica oleracea* is one of the agronomically important species of Brassicaceae family, comprising several economically important vegetables such as cabbage, cauliflower, broccoli, kale, kohlrabi, and brussels sprout. Remarkable morphological diversity is displayed by different morphotypes of *B. oleracea*, which are grown for their leaves, stems, inflorescence, lateral and axillary buds. *B. oleracea* vegetables are healthy and possess a variable amount of vitamin, fiber, minerals, and useful phytochemicals [1,2]. Additionally, *B. oleracea* is a good source of a major class of secondary metabolites, glucosinolates [3], possessing anti-cancer properties. *B. oleracea* production is greatly threatened by a range of fungi, bacteria, viruses and protist of which clubroot disease caused by a root-infecting protist *Plasmodiophora brassicae* Woronin is one of the most destructive diseases affecting *B. oleracea* worldwide. Due to *P. brassicae* attack, an estimated loss of 10% to 15% in both quality and yields of cruciferous crops are reported [4]. Clubroot has caused a loss of 30%, even up to a reduction of 80–91% in yield during experimental field trials in canola [5,6]. Owing to the huge economic impact caused by clubroot, various management strategies have been used to control clubroot disease. However, due to the broad host range of the pathogen and the high survival rate of the resting spores, up to 20 years [7], the management of clubroot becomes complicated. Moreover, traditional cultural practices, including soil liming or fungicides treatment are expensive and unreliable [8]. Crop rotation with non-host species [9,10] and farm machinery sanitization [11] may help in reducing the severity of clubroot to a certain extent. The concentration of resting spores of *P. brassicae* is highly correlated with the severity of clubroot disease [12,13]. Therefore, spore-density reduction could act as an effective strategy to manage clubroot disease. Although various management strategies have been employed to curb clubroot, host-plant resistance offers the most economical, sustainable, and environment-friendly solution to mitigate clubroot disease [14,15]. Planting improved varieties with multiple resistance genes and adopting a crop-rotation scheme would provide a better safeguard against *P. brassicae* growth [16,17]. Hence, an integrated approach involving conventional pest-management strategies and deployment of resistant cultivars could effectively eliminate clubroot disease [16,18,19].

In recent years, the advancement of next-generation sequencing (NGS) and mass spectrometry technologies has provided new insights into the genetic improvement of various crops for biotic and abiotic stress resistance. The omics approaches involving genomics, transcriptomics, proteomics, and metabolomics have enormous potential for understanding the mechanisms of host-pathogen interactions. In the functional genomics era, a holistic approach to using omics technologies has allowed the comprehensive examination of plants and microbes, leading to the discovery of plant defence mechanisms against pathogens. Omics technologies have broader implications in understanding the basic pathogenic mechanisms critical to disease progression. Omics approaches are increasingly applied in Brassica crops to gain insight into the complex mechanisms of host-pathogen interactions. Due to affordable NGS and spectrometry technologies, a considerable amount of data at the multi-omics levels are becoming available in Brassica crops [20], bringing the possibilities of improving disease resistance.

**Figure 1:**
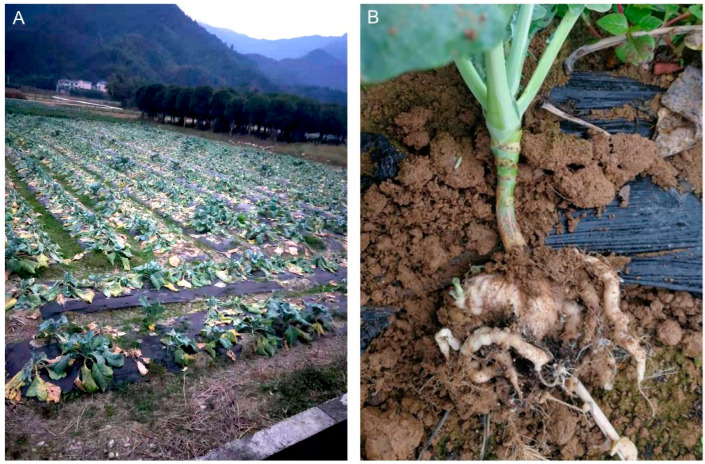
Cauliflower infected with clubroot [(**A**) Cauliflower field heavily infested with clubroot disease, (**B**) Clubroot-infected cauliflower plant with characteristic root gall].

Clubroot, caused by *Plasmodiophora brassicae* Woronin, is a soil-borne and an obligate biotrophic protist and taxonomically belongs to the phylum Plasmodiophoromycota [21]. *P. brassicae* is one of the most devastating diseases infecting Brassica crops [22], reducing quality and yields worldwide [4]. The protist infects the plant through root hairs, which undergo abnormal cell enlargement and uncontrolled cell division, causing the formation of gall, also called ‘club’, which are irregular, nonhomogeneous, and are up to 5 to 6 inches wide (Figure 1). The deformed roots with thick and fleshy growth create a nutrient sink, nourishing the pathogen and leading to cracking and rotting of the roots. The club-shaped roots reduce the efficiency of water and nutrients uptake to the host plants, resulting in stunted growth, chlorosis, leaf abscission, wilting, reduced seed yield, and even the premature death of the infected plants [4,23]. *P. brassicae* has a wide host range that serves as sources of resting spores to perpetuate the infestation cycle [4,24]. Clubroot development is favored by excessive moisture, low soil pH, and soil temperature between 64 to 77 °F. Once the soil becomes infested with clubroot, the spores can survive in the soil as resting spores for up to 20 years, making it difficult to be managed by cultural practices or chemical means [4,25].

*P. brassicae* has a complex life cycle that consists of three stages (i) dormant resting stage, (ii) primary stage (root–hair infection stage), and (iii) secondary zoospore reinfection (cortex infection stage). Under favorable conditions, primary zoospores are released from the resting spores, germinate, and the plant root exudates enhance the germination of the resisting spores [26,27]. The attachment of zoospores to the surface of root hairs initiates primary infection, followed by penetration, and eventually grows to form the primary plasmodia. The primary plasmodia then undergo a series of cell divisions to form intracellular multinucleate secondary plasmodia. The secondary plasmodia directly infect the cortical cells, change the root hormone balance and induce cell hypertrophy (cell division) and cell hyperplasia (cell elongation), resulting in gall formation [28]. The secondary plasmodia are then divided into numerous mature resting spores scattered into the soil when the galls decompose and disintegrate [29], becoming the source of infection for the following year. Once the soil is contaminated with *P. brassicae*, the longevity of the resting spore makes the field unsuitable for the cultivation of Brassicaceae crops.

*P. brassicae* has a wide host range and could infect about 3700 species and 330 genera in the Brassicaceae family [30]. Physiological classification and virulence variation were carried out long ago in the 1930s [31]. *P. brassicae* pathotypes are classified based on the reactions of differential hosts. The accurate estimation of pathotypes of field isolates by host differentials is essential for resistance breeding, as accurate pathotyping assists in selecting resistant cultivars [32]. Based on the virulence pattern, several pathotype classification systems have been proposed in the past decades to study the pathogenic variability. The current differential systems used internationally are Williams’ differential set [33], European clubroot differential (ECD) [34], pathotyping according to Somé [35], Canadian clubroot differential (CCD) [36], and Sinitic clubroot differential set (SCD) [32]. The emergence of new pathotypes is a continuous process, and while testing on the additional hosts, virulence variability could be found within a pathotype [32,37,38].

In *B*. *oleracea*, significant advances were made at the multi-omics level to understand the complex resistance mechanisms of Brassica-*P. brassicae* pathosystem. This review summarizes the impressive contributions of emerging omics technologies to curb clubroot disease in *B. oleracea* by elucidating the dynamic changes transpiring at the metabolic and signaling pathways involved in defence response against *P. brassicae* attack. We have also focused on various aspects of clubroot disease and host-resistance mechanisms at the physiological, biochemical, and molecular levels. Finally, we have discussed the bottlenecks and how to integrate the omics technologies to deliver solutions to breed clubroot-resistant Brassica crops in a holistic, targeted, and precise way.

In the functional genomics era, ‘-omics’ are indispensable, which have been exemplified by many studies in various crops. Therefore, it is required to understand the defence mechanisms of clubroot resistance to establish the theoretical basis of genes and pathways involved in defence response to *P. brassicae* infection at the primary and secondary infection stages. To illuminate this, several ‘-omics’ studies, including genomics, epigenomics, transcriptomics, ncRNAomics, proteomics, and metabolomics have been conducted in Brassica species to elucidate the dynamic changes at the multi-omics levels. In the following sections, this has been discussed in detail to get a comprehensive understanding of the molecular mechanisms of Brassica-*P. brassicae* interaction.

## 2. Genomics

Dramatic innovations in NGS technologies in the last few decades have led to the publishing of whole-genome sequences, millions of molecular markers and high-density genetic maps. Numerous functional genomics studies aimed at characterizing the disease resistance genes may provide insights into the basis of disease resistance [39,40]. The molecular genetic studies in *Brassica* spp. have led to the identification of a large number of QTLs, candidate genes and the allelic variations associated with the resistance genes will accelerate the improvement of *Brassica* spp. against *P. brassicae*.

### 2.1. Pangenome to Identify Novel Resistance Gene Analogs (RGAs)

Reduced sequencing cost has facilitated an increasing amount of reference genome data in *Brassica* spp., including *B. oleracea*. Most of the *B. oleracea* genomes were assembled using short-read sequence technologies by Illumina [41,42]. Recently, third-generation sequencing technologies capable of generating long-read sequences such as single-molecule real-time (SMRT) sequencing (PacBio) and Nanopore sequencing technologies (Oxford Nanopore Technologies, Oxford, UK) have been used to develop high-quality chromosome-scale genome assemblies in broccoli, cabbage, and cauliflower [43,44,45]. But the reference genomes may not represent all the morphotypes of *B. oleracea*, such as brussels sprout (lateral leaf buds) and kohlrabi (tuberous stems) not having genome assemblies. This may result in missing out genetic diversity present within *B. oleracea*, overlooking novel resistance alleles by relying on a single reference genome. This limitation has triggered the development of pangenomes, which allows the identification of comprehensive genomic variations from the gene pools represented by many lines within a species [46]. The pangenome of *B. oleracea* revealed that many R genes are not present in all lines [47] and are dispensable indicating the highly variable nature of R genes. R genes are grouped into a large repertoire called resistance gene analogs (RGAs), including NBS-LRR (TIR-NBS-LRR and CC-NBS-LRR), pattern-recognition receptors (PRRs) comprising receptor-like protein kinases (RLKs), receptor-like proteins (RLPs) and wall-associated kinases [48,49]. Recently, a pangenome study in *B. oleracea* revealed that the wild relative *B*. *macrocarpa* harbors the highest number of (1495) RGA candidates [50], suggesting that wild Brassica could be a large repository of R genes. Using pangenomics, Bayer et al. [50] detected 59 RGA candidates within Sclerotinia, clubroot, and Fusarium wilt resistance QTLs in *B. oleracea*, and the QTLs harbored 28 RLKs. The sequence comparison of the RGAs placed within the QTL region will help candidate-gene identification for disease resistance.

### 2.2. Resistance Sources, Genetics of Resistance and Identification of QTLs for Clubroot Resistance in B. oleracea

Several *B. oleracea* germplasm collections consisting of different morphotypes have been evaluated for clubroot resistance by various research groups, and several resistance sources have been identified. Fewer resistance accessions have been reported in cauliflower [51,52,53,54] compared to other *B. oleracea* varieties such as cabbage, kale, broccoli, brussels sprout, etc. [51,52,55,56,57,58,59]. A large number of accessions of cabbage, cauliflower, broccoli, kale, and brussels sprout were evaluated by Crisp et al. [51] and found resistance in some cabbage and kale accessions. Peng et al. [14] reported two cabbage cultivars, ‘Kilaherb’ and ‘Tekila’, highly resistant to all the five pathotypes (2,3,5,6 and 8) found in Canada. Ramzi et al. [60] evaluated 223 accessions of *B. oleracea* collected from the Netherlands and Canada against different pathotypes [P1, P2, P3, P4, P5, and P1(+)], and only three accessions (CGN11150, CGB14078 and CGN15227) exhibited high resistance to all the pathotypes. Fredua-Agyema et al. [61] screened 65 *B. oleracea* accessions against five pathotypes (2F, 3H, 5I, 6M, and 8N) and 17 other field isolates, and four accessions were resistant, five were moderately resistant, and the remaining 56 were susceptible. In addition, broad-spectrum resistance was exhibited by seven accessions of kale and two accessions of brussels sprout. Farid et al. [62] evaluated 135 *B. oleracea* accessions representing all the eight morphotypes against two pathotypes [F3-14 (3A) and F-359-13 (5X L-G2)], and resistance was observed more frequently in kale. So, among different morphotypes of *B. oleracea*, kale, followed by cabbage, could serve as a potential donor for resistance breeding in *B. oleracea*.

The inheritance of clubroot resistances has been studied using either diallel cross or segregating populations in *B. oleracea*, and continuous resistance including both minor and major genes has been reported. The studies have indicated that recessive genes primarily govern clubroot resistance in *B. oleracea* [55,57,63,64,65,66,67,68,69]. Nevertheless, the dominant nature of resistance has also been reported [70]. The genetic studies also identified additive gene action [71], duplicate genes [64], recessive gene with additive gene action [66,68,69], complementary gene action [72,73] responsible for clubroot resistance in *B. oleracea*. However, most of the classical genetic studies indicated the prevalence of multiple loci controlling clubroot resistance in the C-genome of *B. oleracea* [67,70]. This was again complemented by genetic-mapping studies, suggesting the predominance of the polygenic nature of clubroot resistance in *B*. *oleracea*, discussed later in this section. In contrast, clubroot resistance is mainly controlled by race-specific, major dominant genes in *B. rapa* and *B. napus* (reviewed by Diederichsen et al. [74]).

The rapid accessibility of genomic data, genetic maps, and associated bioinformatics tools has allowed the dissection of the genetic architecture of clubroot resistance in *B. oleracea*. The ‘C’ genome harbors several R genes, and more than 40 QTLs with both major and minor effects have been reported by various authors (Table 1). A mapping study revealed the presence of one broad-spectrum major locus (*Pb-Bo1*) and several isolate-specific and minor QTLs, suggesting the role of both isolate-specific and broad-spectrum QTLs in providing continuous resistance in *B. oleracea* [75]. The quantitative control of resistance in *B. oleracea* was identified by various authors explaining limited phenotypic variance [76,77]. Several studies identified many broad-spectrum QTLs conferring resistance against different isolates of *P. brassicae* [76,78], broadening the spectrum of immunity in *B. oleracea*. The accumulation of five clubroot resistance genes (*Anju1, Anju2, Anju3, Anju4, GC1*) conferred broad resistance against six isolates in *B*. *oleracea*, and the genotype consisting of minor loci (*Anju2, Anju3, Anju4, GC1*) responded in a pathotype-specific manner [76]. This indicated the necessity of both isolate-specific and broad-spectrum resistance to carry out resistance breeding in *B. oleracea*.

For traits with low heritability and polygenic inheritance, especially disease resistance [79], detection of dominant QTLs could be of considerable value for breeding programs. Two dominant QTLs (*CR2a* and *CR2b*) conferring resistance to race 2 of *P. brassicae* in cabbage were mapped on LG 6 and LG 1, respectively [80]. Similarly, three QTLs responsible for resistance to race 7 were identified in an F2 population developed by crossing resistant broccoli with susceptible cauliflower [81]. A multiple QTL mapping approach was used to map two main QTLs (*pb-3* and *pb-4*) in cabbage cv. Bindsachsener on LG3 and LG1 for resistance to *P. brassicae* [82]. Two marker loci, 4NE11a and 2NA8c were closely linked to the resistance loci *pb-3* and *pb-4*, respectively, which would assist in marker-assisted selection for clubroot resistance.

However, the above DNA markers used in mapping were low-throughput, expensive, and time-consuming. After the advent of NGS technologies, the discovery of high-throughput SNP markers in *Brassica* spp. gained momentum, leading to the generation of high-density genetic maps and candidate gene detection in *Brassica* spp. [83,84]. A 60 K bead chip array [77,85] and NGS-enabled genotyping-by-sequencing (GBS) technique [86] have been deployed to discover and screen SNPs for high-density linkage map construction and QTL mapping for *P. brassicae* resistance in *B. oleracea*. In recent years, GBS has emerged as a robust and cost-effective method for improving the resolution of target loci [87], allowing the accurate prediction of candidate genes [88]. A high-density linkage map was constructed using SNP markers developed through GBS technique and two major QTLs (*CRQTL-GN_1* and *CRQTL-GN_2*) for race 9 and a single major QTL (*CRQTL-YC*) for race 2 of *P. brassicae* were identified in cabbage [86]. Utilizing the 60 K Brassica SNP array, a high-density linkage map was constructed and a total of 23 QTLs were identified for disease incidence and clubroot-associated traits in *B. oleracea*, indicating the quantitative nature of clubroot resistance [77]. To provide better mapping resolution, GWAS was implemented and 10 QTLs and six candidate genes were reported in a ±250 Kbp QTL region of *B*. *oleracea* showing significant association with resistance against the two pathotypes (3A and 5X L-G2) [62].

**Table 1:** List of QTLs associated with clubroot resistance in *Brassica oleracea* L.

Species	Mapping Population	Pathotype/Race/Isolate	Gene Locus/QTL	Chr/LG	Reference
*B. oleracea*	F2	Race 2	*CR2a*	LG6	[80]
			*CR2b*	LG1	
*B. oleracea* var. *italica*	F2	Race 7	*14a*	1C	[81]
			*48*	4C	
			*177b*	9C	
*B. oleracea* var. *capitata*	DH	Isolate CD 16/3/30	*pb-3*	LG3	[82]
			*pb-4*	LG1	
*B. oleracea* var. *acephala*	F2	Races 1 and 3	1 *QTL*	LG3	[89]
*B. oleracea* (Kale)	F2:3	Pathotypes P1, P2, P4, and P7	*Pb-Bo1*	LG1	[75]
			*Pb-Bo2*	LG2	
			*Pb-Bo3*	LG3	
			*Pb-Bo4*	LG4	
			*Pb-Bo5a*	LG5	
			*Pb-Bo5b*	LG5	
			*Pb-Bo8*	LG8	
			*Pb-Bo9a*	LG9	
			*Pb-Bo9b*	LG9	
*B. oleracea*	F2	Isolate Kamogawa (races 1 and 3), Anno and Yuki	*QTL1*	LG1	[78]
			*QTL3*	LG3	
			*QTL9*	LG9	
*B. oleracea* var. *capitata*	F2:3	Race 4	*PbBo(Anju)1*	O2	[90]
			*PbBo(Anju)2*	O2	
			*PbBo(Anju)3*	O3	
			*PbBo(Anju)4*	O7	
			*PbBo(GC)1*	O5	
*B. oleracea* var. *capitata*	F2:3	Field isolates—No. 5, No. 14, Ano-01, T-1, I-1, and M-1	*Anju1*	O2	[76]
			*Anju2,*	O2	
			*Anju3,*	O3	
			*Anju4*	O7	
			*GC1*	O5	
*B. oleracea*	F2	Race 4	23 QTLs	C1, C2, C3, C4, C6, C7, C8	[77]
*B. oleracea*	*B. oleracea* accessions	Pathotypes 3A and 5X LG-2	*PbC4.1*	C4	[62]
			*PbC6*	C6	
			*PbC7.1*	C7	
			*PbC7.2*	C7	
			*PbC8*	C8	
			*PbC9.1*	C9	
			*PbC3*	C3	
			*PbC4.2*	C4	
			*PbC7.3*	C7	
			*PbC9.2*	C9	
*B. oleracea* var. *capitata*	F2:3	Races 2 and 9	*CRQTL-GN_1*	C2	[86]
			*CRQTL-GN_2*	C3	
			*CRQTL-YC*	C3	

### 2.3. Fine Mapping and Identification of NBS-LRR Encoding R Genes for Clubroot Resistance

Fine mapping of the resistance genes is necessary to identify candidate genes and successfully facilitate marker-assisted selection (MAS) for disease resistance. Fine mapping of clubroot resistance genes is a success story in *B*. *rapa* owing to the dominant nature of resistance genes, and several resistance genes have been fine mapped (Table 2). Fine genetic and physical mapping have led to the identification of clubroot resistance genes encoding proteins containing the NBS-LRR domain (Table 2). Most of the R genes encode proteins containing NBS-LRR (nucleotide-binding site and leucine-rich repeat) domains [91]. Based on their functional domain, the NBS-LRR protein family is subdivided into N-terminal coiled-coil-domain-containing NBS-LRR (CC-NBS-LRR) and toll/interleukin-1 receptor domain-containing NBS-LRR (TIR-NBS-LRR) subfamilies [92]. Disease resistance genes encoding NBS-LRR-related proteins are effective against hemibiotrophic and obligate pathogens [93]. The specific R genes encoding NBS–LRR proteins can recognize the effector factor secreted by pathogens activating effector-triggered immunity (ETI). Moreover, due to natural selection, pathogens produce new effectors, and host plants evolve to produce new R genes [94].

Saito et al. [95] fine mapped the locus *Crr3* located on chromosome A3 of *B*. *rapa*, and synteny analysis of this region showed homology to the sequences in the top arm of ch. 3 of *A. thaliana*. Similarly, Kato et al. [96] fine mapped a clubroot resistance gene *CRb* in 24.2–24.342 Mb interval on ch. A3 of *B*. *rapa* against pathotype 3. However, the original *CRb* locus mapped by Piao et al. [97] was fine mapped by Zhang et al. [98] to understand the relationship of these clubroot resistance genes. The *CRb* locus fine mapped to a region of approximately 0.14 cM on ch. A3 of Chinese cabbage was tightly linked to two other clubroot resistance genes, *CRa^kato^* and *CRb* (already fine mapped by Kato et al. [96]. In the predicted region of an 83.5-kb interval, 15 putative genes were annotated with potential involvement in clubroot resistance, and the target interval contained one TIR– NBS–LRR (TNL) and one NBS–LRR gene. A new clubroot resistance locus *Rcr1* was fine mapped between 24.26–24.50 Mb on LG A3 in *B*. *rapa* ssp. *Chinensis* [99]. Within this interval, a cluster of four TNL and one defence-related gene was located. Functional annotation of the differential expressed genes (DEGs) showed that genes related to clubroot symptom development were significantly down-regulated in plants carrying *Rcr1.* Gao et al. [100] fine mapped a single dominant clubroot resistance locus to a 187-kbp physical region of ch. A3 in five hybrid Chinese cabbage cultivars and the molecular markers developed could be deployed for marker-assisted selection of clubroot resistance. Recently, fine mapping of the resistance locus *PbBa8.1* within a 2.9 Mb region on ch. A8 was carried out using BSA-Seq [101]. Interestingly, another resistance gene, *Crr1a* was also identified in the same region, suggesting that *PbBa8.1* and *Crr1a* might be allelic but differ in function. Very recently, fine mapping of the clubroot resistance locus *CRA8.1* on chromosome A8 in *B*. *rapa* revealed the presence of two different loci *CRA8.1b* (395 kb fragment) and *CRA8.1a* (765-kb) [102]. While *CRA8.1b* was responsible for the resistance against PbZj and PbXm isolates, together with *CRA8.1a*, it can confer resistance to PbXm+ isolate of *P*. *brassicae*. Two genes encoding TNL proteins and one candidate gene encoding RLP protein were the most probable candidate genes responsible for clubroot resistance in DingWen (DW). Again, the same locus *PbBa8.1* was fine mapped in the ECD04 (most valuable resources for CR breeding) genome and narrowed down into a 1428 Kb region and nine candidate genes were identified in this region [103]. Functional analysis of one candidate gene, *CRA8.2.4*, with NBS-LRR domain revealed that the plants with higher expression of *CRA8.2.4* were not infected with clubroot. In contrast, the plants with lower or no expression showed a higher disease index.

BSR-seq, combining BSA and RNA-seq techniques, has been used in *Brassica* spp. to determine QTLs, fine mapping and reporting of candidate genes for clubroot resistance encoding TNL resistance proteins [104,105,106,107,108,109]. Using BSR-seq, a major gene, *Rcr5* was fine mapped into the 23–31 Mb region of the A3 chromosome, and the identification of several recombinants indicated that *Rcr5* was different from the previously mapped clubroot resistance genes (*CRa*/*CRb^kato^*, *CRb*) on ch. A3 [108]. Another clubroot resistance gene, *Rcr2* was identified and fine mapped on ch. A3 using BSR-Seq in Chinese cabbage cv. “Jazz” in an interval of 0.4 cM and five SNP markers co-segregating with *Rcr2* were identified [105]. In the target region, four genes encoding TNL proteins were detected among which two genes with high numbers of polymorphic variants could be the most likely candidates for *Rcr2*. In *B*. *oleracea*, a major clubroot resistant gene, *Rcr7* was mapped to a physical interval of 41–44 Mb on chromosome 7, exhibiting resistance against two pathotypes (Pathotype 3 and 5X) through the BSR-seq approach [106]. Seven clubroot resistance genes placed in the target region encoding TNL resistance proteins were identified. 

In the post-genomics era, advent of NGS technologies has enabled the discovery of high-throughput SNP markers leading to high-density linkage map construction, NGS-enabled BSA-seq techniques etc. This has helped in the rapid identification of candidate clubroot genes containing the TIR-NBS-LRR domain in *B*. *oleracea* [110], *B*. *rapa* [111,112,113,114,115] and *B*. *napus* [116,117]. To rapidly identify the candidate genes for clubroot resistance, Ce et al. [110] employed a QTL-seq approach to identify four QTLs (*qCRc7-1*, *qCRc7-2*, *qCRc7-3*, and *qCRc7-4*) on ch. C7 and one on C4 (*qCRc4-1*) in *B. oleracea*. Out of 312 genes located in the three QTL regions (*qCRc7-2*, *qCRc7-3*, and *qCRc7-4*) on C7, six R genes encoded TNL proteins. Further, two potential candidate genes [Bol037115 (FLZ domain protein) and Bol042270 (plant intracellular Ras-group-related LRR (PIRL) protein] were identified and a functional marker “SWU-OA” was developed, exhibiting 95% accuracy in identifying clubroot-resistant lines. Though several clubroot resistance genes containing the NBS–LRR domain have been reported, the role of NBS-LRR proteins in activating ETI for disease resistance is not clearly understood in *Brassica*–*P*. *brassicae* pathosystem.

While the LRR domain of TIR-NBS-LRR proteins is the primary determinant of recognition specificity of the effector (Reviewed by Collier and Moffett [118]), the TIR domain is involved in cell-death induction [119]. The deletion of the TIR domain resulted in clubroot susceptibility of *Crr1a^A9709^* allele in *B*. *rapa* [120] and site-directed mutagenesis in the TIR domain identified loss-of-function of TNL gene [119]. To date, only three clubroot resistance genes, *CRa* (ch. A3) [121], *Crr1a* (ch. A8) [120], and *CRb* (ch. A3) [122] have been cloned in *B*. *rapa*. These cloned genes have been characterized as classical resistance genes, among which *CRa* and *Crr1a* encode TNL and *CRb* contains the NB-LRR domain. Interestingly, the DNA sequence of the functional ORF of *CRb* gene was found to be identical to the previously cloned *CRa* gene, suggesting that *CRb* and *CRa* are one and the same resistance gene [122]. However, the resistance mechanisms associated with these three genes have not been clarified yet. Recently, gain-of-function analysis using the chimeric genes revealed that 172 amino acids in the C-terminal region are essential for clubroot resistance imparted by the *Crr1a* gene [123]. Furthermore, very recently, one candidate gene, named *CRA3.7.1*, identical to *CRa* from *B*. *rapa* T136-8 [121] and *CRb*_α [122] was functionally characterized by Yang et al. [103]. *B*. *napus* lines with stable transformation with *CRA3.7.1* were resistant against different Chinese isolates (pathotype 4) of clubroot. However, unlike *B*. *rapa*, the complex inheritance pattern and quantitative nature of resistance in *B*. *oleracea* [86,124] have made it difficult to fine map/clone the clubroot resistance genes restricting the deployment of R genes in resistance breeding.

**Table 2:** List of clubroot resistance genes with NBS-LRR domain in *Brassica* spp.

Species	Mapping Population	Pathotype/Race/Isolate	R Gene	Chr/LG	Fine Mapped Region/Interval	Reference
*B. oleracea*	BC1	Pathotype 3 and 5X	*Rcr7*	C7	41–44 Mb	[106]
*B*. *oleracea*	F2	Race 4	*qCRc7-1*	C7	38.33–44.14 Mb	[110]
			*qCRc4-1*	C4	16.92–18.79 Mb	
			*qCRc7-2*	C7	38.96–39.54 Mb	
			*qCRc7-3*	C7	41.38–42.52 Mb	
			*qCRc7-4*	C7	43.56–44.15 Mb	
*B. rapa*	F1	Field isolate (PbZj, PbXm, PbXm+)	*CRA8.1a*	A8	765 kb segment	[102]
			*CRA8.1b*	A8	395 kb segment	
*B. rapa*	BC1	Pathotype 3	*Rcr2*	A3	0.4 cM (~250 kb) segment	[105]
*B. rapa*	F2	Pathotype 3	*Rcr5*	A3	23–31Mb segment	[108]
*B. rapa*	BC3F2	Pathotype 4	*PbBa8.1*	A8	2.9 Mb segment	[101]
*B. rapa*	F2	Race 2	*CRa*	A3		[121]
*B. rapa*	F2	Isolate Ano-01 (Pathotype 4)	*Crr1a*	A8	8 kb segment	[120]
*B. rapa*	F2	Pathotype 3	*CRb^kato^*	A3	24.2–24.342 Mb	[96]
*B. rapa*	F2	Pathotype 4	*CRb*	A3	0.14 cM segment	[98]
*B. rapa* ssp. *chinensis*	BC1	Pathotype 3	*Rcr1*	A3	24.26–24.50 Mb	[99]
*B. rapa*	F2:3	Isolate Ano-01	*Crr3*	A3	0.35 cM segment	[95]
*B. rapa*	F2	Pathotypes 2, 5 and 6	*Rcr1*	A3		[104]
*B. rapa*	BC1	Pathotypes 3H and 5X	*Rcr3*	A8	10.00–10.23 Mb	[109]
			*Rcr9^wa^*	A8	10.85–11.17 Mb	
*B. nigra*	F2	Pathotype 3	*Rcr6*	B3	6.1–6.6 Mb	[107]
*B. rapa*	F2, BC	Pathotype 3	*Rcr6*	A8	14.8–15.4 Mb	[107]
*B. rapa*	BC1	Pathotypes 2, 3, 5, 6, 8	*Rcr4*	A3	2.96 Mb segment	[111]
		Pathotype 5x	*Rcr8*	A2	3.59 Mb segment	
			*Rcr9*	A8	6.48 Mb segment	
*B. rapa*	DH	Pathotype “Banglim”	*PbBrA08^Banglim^*	A8	~1 Mb segment	[114]
*B. rapa*	F2	Pathotype 4	*CRs*	A8	0.8 Mb segment	[113]
*B. rapa* ssp. *Pekinensis*	F2	Race 4	*CRd*	A3	60 Kb segment	[112]
*B. napus*	DH	Field isolate (Lower Silesian Province, Poland)	*Crr3^Tsc^*	A3	0.4 cM segment	[116]
*B. napus*	DH	Pathotypes 3A, 3D, 3H	*Rcr10^ECD01^*	A3	3.7 Mb segment	[117]
		Pathotypes 3A, 3D, 3H, and 5X	*Rcr9^ECD01^*	A8	2.2 Mb segment	

### 2.4. Pyramiding of Resistance Genes

Resistance conferred by single genes is not durable and prone to break down. Pyramiding clubroot resistance genes with different resistance mechanisms could be a good strategy for accumulating multiple resistance genes into a single cultivar. Pyramiding multiple R genes or combining genes with minor effects have improved the durability of resistance [125,126]. The accumulated genes may impart resistance to either multiple pathotypes [127,128] or higher resistance to a single pathotype [76] of *P*. *brassicae*. Previously, the breakdown of clubroot resistance has been reported in single-gene homozygous lines of Chinese cabbage [127] and *B*. *napus* [129]. A resistant canola cultivar reportedly showed increased clubroot severity to pathotype 3 of *P*. *brassicae* under controlled conditions [130]. Matsumoto et al. [127] pyramided three major clubroot genes (*CRa*, *CRk*, and *CRc*) and developed a homozygous Chinese cabbage line conferring higher resistance to all the six isolates of *P*. *brassicae*. Similarly, Shah et al. [128] pyramided clubroot resistance genes, *CRb* (resistant to race 2, 4, and 8) and *PbBa8.1* (resistant to pathotype 4) in *B*. *napus*, and developed homozygous and heterozygous lines through MAS. Comparatively, homozygous lines exhibited better resistance than the heterozygous lines, suggesting that the accumulation of multiple resistance genes into a single line in a homozygous state could lead to durable resistance. However, in *B*. *oleracea* few attempts have been made to pyramid the clubroot resistance genes for broad spectrum and durable resistance [76,78]. Tomita et al. [76] illustrated the single and cumulative effects of clubroot resistance gene(s) by evaluating the resistant lines carrying multiple combinations of clubroot resistance genes against different isolates of *P*. *brassicae*. Incidentally, a single involvement of the major resistance gene located in *PbBo (Anju)1* or accumulation of minor QTLs did not provide sufficient resistance. However, the genotype with a combination of a major resistance gene with all the four minor QTLs imparted the highest resistance against the six isolates of *P*. *brassicae*. Hence, for successful resistance breeding, the accumulation of both major and minor clubroot resistance genes is necessary for durable resistance against multiple pathotypes of *P*. *brassicae* in *B*. *oleracea*.

### 2.5. Comparative Mapping Studies

During evolution, diploid Brassica species have evolved from a common hexaploid ancestor [131,132,133,134] through whole-genome triplication of the ancestral genome, leading to reshuffling and changes in the genome number [134,135,136]. Comparative linkage mapping has elucidated the degree of genomic conservation through micro and macro synteny studies, and conserved genomic regions have been identified in Brassica species [137,138,139,140]. Gene conservation and microsynteny analysis of *B*. *napus* with *B*. *rapa* and *B*. *oleracea* reported high conserved collinearity with their orthologues across the genome, suggesting little or no changes in the microstructure of specific segments during hybridization events [141]. *B*. *oleracea* genome (CC) shares a high ancestral synteny with the genome (AA) of *B*. *rapa* with a high level of conserved gene content and order [41,142]. Both the genomes share a set of 24 conserved chromosomal blocks [143].

Comparative mapping may exploit the conserved synteny between *B*. *rapa* and *B*. *oleracea* genome to reveal the relative positions of the clubroot-resistant QTLs in *B. oleracea* [77,86,90]. Synteny analysis of *B*. *oleracea* with previously reported QTLs of *B*. *rapa* [95,96,144,145,146,147] revealed strong collinearity of *CRQTL-GN_1* located on ch. C2 with ch. A2 of *B*. *rapa* harboring *CRc* gene where its linked marker ‘m6R’ was mapped [86]. Comparative analysis by Nagaoka et al. [90] also showed that a minor (*pb-Bo (Anju)2*) and major QTL (*pb-Bo (Anju)1*) located on C2 of *B*. *oleracea* are collinear with the genomic region of A2 of *B. rapa* carrying *CRc* gene. These results indicated that *pb-Bo (Anju)2* and *pb-Bo (Anju)4* loci were homologous to *CRc* and *CRb* genes of *B*. *rapa*, respectively. A QTL interval overlapped by NFR.II-3 and PCR.II-2 on C3 (6.28–6.32 Mb) was partially syntenic to A2 chromosome (0.59–6.22 Mb) of *B*. *rapa* where the marker, m6R linked to *CRc* [147] and *Pb (Anju)2* [90] was located [77]. Another QTL (NFR.II-7) on C7 was syntenic to a region overlapped with fine-mapped clubroot resistance loci, *Crr1* on A8 of *B*. *rapa*. Two expressed and unexpressed TNL genes located in QTL *Rcr7* on ch. C7 were homoeologous to four *B*. *rapa* genes residing in a 25 Mb region of ch. A3, which were different from the previously mapped resistance genes (*Rcr1/Rcr2/Rcr4/CRa/CRb*) of *B*. *rapa* [106]. Hence, *Rcr7* mapped in *B*. *oleracea*, could have originated from a gene in ch. A3 of *B*. *rapa*, which is different from *Rcr1/Rcr2*/*Rcr4*.

*Brassica* spp. and *Arabidopsis thaliana* have originated from a common ancestor, so certain chromosomal segments are expected to be conserved. Microsynteny analysis has suggested that each genomic segment of *A*. *thaliana* corresponds to three syntenic copies of *B*. *rapa* [148]. Clubroot resistance genes have originated from major resistance clusters (MRCs) of a common ancestral genome and distributed to different regions during the evolutionary process [146,149]. The regions harboring clubroot resistance genes in *B*. *oleracea* correspond to MRCs of *A*. *thaliana* genome. The regions of clubroot resistance QTLs, *pb-Bo (Anju)1* and *pb-Bo (Anju)3* corresponded to the top of ch. 5, *pb-Bo (Anju)2* and *pb-Bo (Anju)4* corresponded to the middle of ch. 4 and *pb-Bo (Anju)4* corresponded to the distal end of ch. 1 of *A*. *thaliana* [90]. In *B*. *rapa*, clubroot resistance genes, *Crr1*, *Crr2*, and *CRb* were in synteny with the central region of ch. 4 of *A*. *thaliana* [124,146]. The regions of *Crr3* of *B*. *rapa* corresponded to the ch. 3 of *A*. *thaliana* [95]. The comparative analysis of clubroot resistance genes among various species of Brassicaceae indicated that clubroot resistance genes originated from a common ancestor and are highly conserved across all the lineages of Brassicaceae family.

Over the past 20 years, good progress has been made in mapping many promising clubroot-resistant genes/QTLs in the A-genome in *B*. *rapa* (Supplementary Table S1). Two resistance loci, *CRa* and *CRb^kato^* are allelic since they are localized in the same position [122], while *Rcr1*, *Rcr2*, and *Rcr4* were found co-localized with *CRa*/*CRb^kato^* [104,105,111] on A3 chromosome of *B*. *rapa*. Additionally, three clubroot resistance hotspots corresponding to *Crr3*/*CRk*/*CRd* and *CRa*/*CRb*/*CRb^kato^* regions on A3 and *Crr1* region on the ch. A8 in *B*. *rapa* were identified [150]. In *B*. *napus*, more than 30 clubroot resistance loci have been mapped in the AC genome against several *P*. *brassicae* strains (Supplementary Table S1). Most of the loci (19 QTLs) mapped by Werner et al. [151] are race-specific and spread across eight chromosomes. In *B*. *napus*, the major resistance gene of the A genome [74,152,153] have been used to develop clubroot-resistant *Brassica* oilseeds. Hence, for sustainable production of *B*. *napus*, it is essential to broaden the genetic base of clubroot resistance by identifying novel resistance in the C genome of *B. oleracea*.

## 3. Epigenomics

Due to the rapid evolution of plant pathogens, mere exploitation of the resistance (R) genes through classical breeding approaches may be less effective [154]. Moreover, there is a gradual erosion of the diversity of R genes owing to intense artificial selection in conventional breeding practices. In recent years, the breeders have been exploiting the epigenetic variation through epi-breeding, which has provided a new avenue for crop improvement [155,156,157]. Epigenetic variations through natural diversity or artificially induced variation (by chemical treatments, induced gene-specific DNA methylation, and epigenome editing) have shown great potential in regulating crop-disease resistance. The epigenetic processes are highly interconnected and orchestrate defence-related transcriptional programming for generational and transgenerational defence priming and respond to pathogen infections [158]. The merger of epigenetics with genomics, known as epigenomics, has developed as a new omics technique to understand the epigenomic molecular mechanisms underlying plant-disease resistance. There is increasing evidence of epigenetic processes governing transcriptional regulation of plant defence responses against a wide range of phytopathogens [159,160,161]. So, as an alternative approach, epigenetics, including DNA (de)methylation, post-translational modification (PTM) of histones, chromatin assembly and remodeling, and RNA methylation, could broaden the phenotypic diversity, essential for crop improvement for disease resistance. DNA methylation usually refers to the addition of a methyl at the C5 position of cytosine base to form 5-methylcytosine (5 mC) and is considered an important epigenetic modification in regulating gene expression and transposon silencing [162,163]. With the rapid development of high-throughput sequencing, several advanced DNA methylation profiling techniques such as whole-genome bisulfite sequencing (WGBS), shotgun bisulfite sequencing, methylation-sensitive amplified fragment length polymorphism (MSAP), methylated DNA immunoprecipitation sequencing (MeDIPseq), methyl-CpG binding domain protein capture sequencing (MBDCap-seq), etc., have been used in DNA methylome analysis in model crop plants [164,165,166,167]. Using the MSAP technique in 30 *B*. *oleracea* populations and lines, Salmon et al. [168] reported high DNA methylation levels and polymorphism related to high structural genome plasticity. As a part of plant defence response, pathogen-infection-induced, DNA hypomethylation in many plant species, including *A*. *thaliana*, resulted in elevated pathogen resistance [169]. DNA methylation studies have been carried out in *Brassica* spp. to reveal the plant’s immunity against pathogens. A recent study by Tirnaz et al. [170] has shown that DNA methylation regulates the promoters of defence genes, causing variation in resistance response against black leg infection at the seedling stage of *B*. *napus*. The resistant cultivar exhibited a higher number of differentially methylated defence-gene promoters than the susceptible cultivar. Very recently, whole-genome DNA methylation analysis in *B*. *rapa* revealed the potential regulatory role of DNA methylation modification in the defence mechanism against white rust [171]. The heritable differences in DNA methylation, called transgenerational epigenetic variants, also contribute to quantitative disease resistance. This can be achieved by developing epigenetic recombinant inbred line (epiRIL) populations with the similar genotypic constitution but different DNA methylation levels at specific chromatin regions. An epiRIL population was constructed in *A*. *thaliana* to decipher the epigenetic architecture of clubroot resistance. A total of 16 epiQTLs were detected, and six epiQTLs were co-localized with previously identified clubroot resistance QTLs, indicating that quantitative resistance to clubroot is mediated by a combination of both allelic and epiallelic variations [172]. Generally, methylation levels vary according to the genomic location and transposon-enriched regions possess a comparatively higher methylated level than the genetic regions [164]. Hence, transposon elements associated with DNA methylation could contribute to pathogen resistance and susceptibility [163]. Another important epigenetic mechanism, post-translational modification (PTM) of histones, such as acetylation, methylation, and ubiquitylation, usually occur at the N-terminal tails of histone, thereby regulating the chromatin structure and function [159,161,173]. The involvement of histone (de)acetylation, methylation and ubiquitylation in regulating plant-pathogen interactions in different crops have been reviewed extensively by Zhi and Chang [158]. Chromatin assembly and remodeling also regulate plant disease resistance. Chromatin structure modulates DNA accessibility to transcription machinery and plays a very important role in gene transcription regulation [174]. Several studies have revealed the involvement of chromatin structure in regulating plant defence responses [173,175,176,177]. Hence, the breeders need to go beyond the conventional breeding strategy of depending on genetic variation and instead, they look towards a wider perspective on how epigenetic modification might regulate plant immunity against pathogens in Brassica. Though the study of plant epigenetics has a long and rich history, only recently, an epigenetic map was constructed for the first time in *B*. *napus* [178]. Based on MSAP, retrotransposon- and centromeric-specific markers, QTLs associated with important agronomic traits in the centromeric regions of five linkage groups were identified. Thus, in *Brassica* spp., limited epigenetic studies have been published and warrant further investigation to decipher the role of epigenomics in understanding the mechanism of Brassica-pathogen interaction. This will fill the critical knowledge gap and can be a solution to the existing limitations to minimize yield loss due to plant disease.

## 4. Transcriptomics

### 4.1. Hormone Signal Transduction during Brassica-P. brassicae Interaction

*P*. *brassicae* is a biotroph and is required to be co-cultured with its host, making it difficult to decipher the molecular mechanisms of Brassica-*P*. *brassicae* interactions. Nevertheless, in recent years, several RNA-seq studies were framed in *B*. *oleracea* to reveal the mechanisms of clubroot resistance by uncovering the genes involved in host defence responses (Table 3). *P*. *brassicae* infection can modify plant hormone homeostasis, leading to clubroot formation [179,180,181,182]. Comparative transcriptome analysis between clubroot-susceptible broccoli inbred line and resistant wild cabbage revealed the down-regulation of DEGs associated with plant hormone signal transduction at the secondary infection stage, suggesting that the defence responses are induced in the primary stage, and are repressed at the secondary infection stage [183]. Similarly, through transcriptome analysis, Wang et al. [184] identified resistance-related genes involved in plant hormone signaling playing important roles at early stage of *P*. *brassicae* infection, also reported by Chen et al. [185].

The hormonal homeostasis of auxin and cytokinin play pivotal roles during host–*P. brassicae* interactions, triggering hypertrophy and hyperplasia, resulting in clubroot formation [22,186,187,188,189]. The hypertrophies of clubroot infected plants go hand in hand with the changes in homeostasis of auxin and cytokinin, which are host and time-dependent [22,188]. Microarray analysis and laser microdissection [190] have confirmed the role of auxin and cytokinin metabolism and signaling in clubroot formation in Arabidopsis. The ability of the plants to maintain auxin and cytokinin homeostasis may help control clubroot formation in the infected root tissue [191]. During clubroot development, auxins accumulate and increase in the infected tissue in a sink-like manner [22]. Xu et al. [192] suggested that activation of IAA signaling stimulates the root hair infection at the early stage of *P*. *brassicae* infection in *B*. *napus*. The auxin biosynthesis gene “nitrilase” mutants delayed gall formation and reduced gall size in *A*. *thaliana* [193,194]. The up-regulation of genes involved in auxin homeostasis such as nitrilases (*NIT1* and *NIT2*), auxin-induced *GH3* homologs, and putative auxin transport proteins were exhibited in *A. thaliana* during clubroot development. In contrast, genes involved in cytokinin homeostasis were down-regulated at an early stage of infection [179]. However, Zhao et al. [195] reported the up-regulation of auxin and cytokinin biosynthesis-related genes at a very early phase of infection (24 hai), demonstrating early regulation of both growth hormones during clubroot development. On the contrary, down-regulation of host auxin biosynthesis and transport-related genes at the later stage of infection was consistent with reduced gall formation in resistant wild cabbage line [183]. *GH3* and *SAUR* are important in decreasing IAA content, thereby relieving the expansion of root cells. Auxin-induced GH3 family conjugates IAA to several amino acids and reduces IAA concentrations [196]. *SAUR* genes negatively regulate auxin synthesis and transport [197] and the expansion of root cells. Ning et al. [198] reported the up-regulation of six GH3 (encodes indole-3-acetic acid amido synthetase) and one *SAUR* gene in clubroot-resistant cabbage cultivar (XG). Similar results of up-regulation of most of the auxin-related genes (ARFs, IAAs, GH3, *SAUR*) in symptomless roots (SL) of kohlrabi were also reported by Ciaghi et al. [189].

Cytokinins play a vital role in gall development by regulating cell division and increasing the availability of nutrients by interfering in the sugar metabolism and invertase production [22,179,180]. The root-protein profile of a susceptible canola genotype revealed the crucial role of cytokinin in the early phases of clubroot infection [199]. Though cytokinin increases in the early response to *P*. *brassicae* infection [181,200], it was reportedly down-regulated at later stages of gall development [180,181]. However, Laila et al. [201] reported the induction of several cytokinin biosynthetic and signaling genes at early and late stages of infection in both root and leaf tissues of Chinese cabbage. Most of the cytokinin genes, including cytokinin dehydrogenase/oxidase family genes (CKX5), CK receptors, CK-regulated UDPglucosyl transferase, and cytokinin synthesis genes except CKX6, were up-regulated in symptomless roots of kohlrabi [189]. This could be possible as, in *A*. *thaliana*, overexpression of CKX has reduced gall formation [179], indicating that more active CK metabolism in the SL root tissue might interfere with clubroot. Similar results were also obtained by Zhu et al. [202], who reported significant up-and down-regulation of three CKX genes (*Bol020547, Bol028392*, and *Bol045724*) in resistant and susceptible cabbage, respectively. Cytokinin response factor 4 gene (*CRF4*) is a component of cytokinin signaling pathway positioned close to clubroot resistance loci *Crr1a and CRa* was reported to mediate resistance against clubroot in *B*. *napus* [203].

A pathogen attack during active growth triggers the plants to execute efficient and systemic defence mechanisms by recognizing particular signals from the damaged cells to initiate defence response [204,205]. Through different kinds of local, constitutive, and inducible mechanisms, plants protect themselves against invading pathogens [206]. Multiple signal-transduction pathways such as SA, ET, and JA pathways stimulate the defence responses and induce several forms of systemic resistance, such as systemic acquired resistance (SAR) and induced systemic resistance (ISR) [207,208]. In plants, phytohormones salicylic acid (SA) and jasmonic acid (JA) are the crucial central defence signaling molecules playing vital roles against biotrophic and hemibiotrophic pathogens. SA is involved in plant defence against biotrophic pathogens, whereas JA and ethylene (ET) act against necrotrophic pathogens [209]. Abscisic acid (ABA) and ethylene have been reported to antagonize or synergize the SA and JA signaling pathways [210,211,212], indicating hormonal cross-talk playing crucial roles to optimize the immune response. A proteomic study at the initial infection stage in *B*. *rapa* revealed the involvement of proteins in SA-mediated SAR, JA/ET-mediated ISR against *P*. *brassicae* [213]. Exogenous application of SA and JA has significantly reduced root gall formation in *B*. *oleracea* and Arabidopsis during *P*. *brassicae* attack [214,215]. In *B. rapa*, higher expression of effectors receptors and PR genes involved in SA signaling in the resistant plant indicated a robust ETI response as part of an early defence mechanism [185]. SA is involved in activating SAR in plants [216]. This was evident as sid2 (salicylic acid induction-deficient 2) Arabidopsis mutants with SAR-deficient npr1-1 and SA-deficient isochorismate synthase 1 (*ICS1*) showed increased susceptibility to *P*. *brassicae* compared to the resistant bik-1 mutant with elevated SA levels [217]. *NPR1* (nonexpressor of PR gene 1) genes and *ICS1* are the key regulators of SA signaling and biosynthesis in plants [93]. Upon *P*. *brassicae* inoculation, two *NPR1* homologs were up-regulated in clubroot-resistant *B*. *oleracea*, leading to downstream SA signaling activation [183]. One TGA factor (responsible for SA-dependent interaction), *TGA4* was up-regulated, and *TGA1* was weakly down-regulated in the resistant genotype, suggesting that *TGA4*-*NPR1* interaction induces SA-dependent pathway in the resistant genotype. Similar results were obtained by Ning et al. [198], who reported the up-regulation of *NPR1* transcript and *TGA4* (interact with *NPR1* to induce SAR) in the resistant line (XG) of *B*. *oleracea*. However, *NPR1* and *ICS1* were not up-regulated significantly in cabbage at the early stage of infection, suggesting that SA signaling pathway-related genes might be involved in resistance at the later stage of infection [184]. Up-regulation of genes related to SA signal transduction has been reported at the secondary infection stage in *B*. *oleracea* [198]. Nevertheless, SA alone is not sufficient to instigate the defence response against *P*. *brassicae* [218]. With an increase in SA levels in clubroot tissue, a SABATH-type methyltransferase (*PbBSMT*) secreted by *P*. *brassicae* [211,219] methylates SA (MeSA) leading to its removal from local infected tissue, thereby disrupting the SA-induced host defence pathway, also reported by Ludwig-Müller et al. [220] and Ciaghi et al. [189].

In general, a higher accumulation of JA is shown in clubroot susceptible hosts and reduced in resistant genotypes [182,221]. Accumulation of JA happens in the developing root galls [222], and several JA-responsive genes were induced in the infected root tissues of clubroot susceptible *A*. *thaliana* [179,222]. Jasmonate resistant 1 (jar1) mutant lines of Arabidopsis with impaired JA-Ile accumulation showed higher susceptibility to *P*. *brassicae* [222]. Enhanced expression of the *BnMYB28.1* gene (regulate clubroot development by modulating aliphatic GSL metabolism) was observed by the exogenous treatment of JA during *P*. *brassicae* infection in *B. napus* [223]. JA also induces the enzymes involved in indole GSLs and auxin biosynthesis, which generally increases during clubroot infection [224]. Enzymes involved in indole GSLs biosynthesis (TrpOxE) and catabolism (myrosinase, nitrilase) were enhanced after treatment with JA and methyl jasmonate, suggesting that increased JA level during clubroot development may up-regulate these three enzymes, thereby enhancing indole GSLs and auxin biosynthesis [225]. Ciaghi et al. [189] reported the down-regulation of JA synthesis in symptomless roots of *B*. *oleracea*. JAZs (Jasmonate ZIM domain-containing protein) act as the key transcriptional repressors in JA signaling pathway [226]. Several JAZs were up-regulated in the resistant genotype of *B*. *oleracea*, though significantly down-regulated in the susceptible genotype [198,227]. This proves that repression of the JA signaling pathway happens in the resistant genotype during *P*. *brassicae* infection. However, several exceptions have been reported where JA signaling mediates resistance against *P*. *brassicae* in *B*. *rapa* [99,228]. In summary, auxin and cytokinin signaling play a significant role in the development of clubroot disease and root gall. Both the hormones stimulate division and elongation of the host cell of the infected- roots during clubroot formation. Similarly, JA signaling primarily enhance clubroot susceptibility with the induction of JA-responsive genes in the clubroot-infected roots. In contrast, SA plays a central role in regulating immune response against *P*. *brassicae* infection by reducing root gall formation significantly.

**Table 3:** Published transcriptomic and differential gene expression studies in *Brassica* spp. on clubroot resistance.

Species	Pathotype/Race/Isolate	Target Tissue	Time Point (Tissue Collection)	Inference	Reference
*B. oleracea var. italica* and *B. macrocarpa*	Pathotype 4	Roots	0, 7, and 14 dai	Genes related to NBS-LRR proteins, SA signal transduction, cell wall and phytoalexins biosynthesis, chitinase, Ca^2+^ signaling and RBOH were up-regulated in the resistant genotype	[183]
*B*. *oleracea* var. *capitata*	-	Roots	7 and 28 dai	Six *BoSWEET* genes were up-regulated in the susceptible cultivar	[229]
*B*. *oleracea* var. *capitata*	-	Roots	7 and 28 dai	22 BoSTP genes harbouring the conserved sugar transporter domain were identified. *BoSTP4b* and *BoSTP12* were involved in monosaccharide unloading and carbon partitioning associated with *P*. *brassicae* colonization	[230]
*B. oleracea* var. *capitata*	16/4/0 (ECD)	Roots	3 dai	Clubroot resistance genes were involved in pathogen recognition, cell wall modification, plant hormone signaling, generation of ROS, transcriptional regulation	[184]
*B.oleracea* var. *gongylodes*	Field isolate (Ranggen, Austria)	Roots	-	Genes involved in host cell wall synthesis and reinforcement, cytokinin metabolism and signaling, SA- mediated defence response were up-regulated and jasmonic acid synthesis was down-regulated in symptomless roots	[189]
*B*. *oleracea* var. *capitata*	Pathotype 4	Roots	7 and 28 dai	Genes associated with cell-wall modification, PRRs, disease resistance proteins, SA signal transduction, calcium influx, RBOH, MAPK cascades, transcription factors and chitinase were up-regulated in the clubroot-resistant line	[198]
*B. oleracea* var. *capitata*	Pathotype 4	Roots	28 dai	Most of the JAZs (Jasmonate ZIM) were activated in the resistant line	[227]
*B*. *oleracea* var. *capitata*	Race 4	Roots and leaves	0, 7, 14, 21, 35, 42, 52 and 60 dai	*MES* genes are important to control clubroot disease. SA biosynthesis is suppressed in resistant plants	[231]
*B*. *oleracea*	Race 4	Roots	7 and 28 dai	36 *CKX* genes were identified and three genes were down- and up-regulated significantly in the susceptible and resistant materials, respectively	[202]
*B*. *oleracea*	Isolate yeoncheon	Leaves and roots	0, 3, 6, 12, 24, 72 hai and 15 and 40 dai	Two *BolMyro* and 12 *BolMBP* genes were highly expressed in the susceptible line, whereas only one *BolMyro* and five *BolMBP* genes were highly induced in the resistant line	[232]
*B*. *rapa* ssp. *pekinensis*	Pathotype 4	Total root tissue	0, 2, 5, 8, 13, and 22 dai	Genes associated with auxin, PR, disease resistance proteins, oxidative stress, and WRKY and MYB transcription factors were involved regulating clubroot resistance	[233]
*B*. *rapa*	Pathotype 4	Roots	10 dai	Brassica-specific genes (BSGs), which are expressed in various tissues can be induced by *P*. *brassicae*	[234]
*B*. *rapa*	Pathotype 3	Total root tissue	15 dpi	In the resistant plant carrying *Rcr1*, genes related to jasmonate and ethylene metabolism, signaling and biosynthesis of callose and indole-containing compounds were up-regulated	[99]
*B*. *rapa*	Pathotype 4	Roots	0, 12, 72, and 96 hai	Genes associated with effector receptors and PR genes involved in SA signaling pathway were induced in clubroot-resistant NIL conferring *CRb-mediated* clubroot resistance	[185]
*B. rapa* ssp. *pekinesis*	Pathotype 4	Roots	30 dpi	DEGs related to metabolic process, biological regulation, calcium ion influx, glucosinolate biosynthesis, response to stimulus, plant-pathogen interaction, plant hormone signal transduction, cell wall thickening, SA homeostasis, chitin metabolism, pathogenesis-related pathways showed significant upregulation in the resistant line	[188]
*B. rapa*	Pathotype 4	Roots	0 h and 4 and 8 dpi	Resistant line carrying the *CRd* gene revealed that plant hormone signal transduction (SA, JA, ET, and BR) played key roles in the late stages of *P*. *brassicae* infection	[228]
*B*. *rapa* var. *pekinensis*	Pathotype 4	Roots and leaves	1, 3, 14, 28, and 35 dai	*BrGH3.3* and *BrNIT1* (auxin signaling), *BrPIN1* (auxin transporter), *BrDCK1* (auxin receptor) and *BrLAX1* (root hair development) were involved in auxin signaling	[235]
*B*. *rapa* ssp. *chinensis*	Pathotype 3	Roots	15 dpi	Increased biosynthesis of lignin and phenolics play a major role in defence responses	[236]
*B*. *rapa* var. *pekinensis*	Pathotype 4	Roots and leaves	1, 3, 14, 28 and 35 dai	cytokinin related genes (*BrIPT1*, *BrRR1*, *BrRR3* and *4)* up-regulated during gall enlargement	[201]
*B*. *rapa* ssp. *pekinensis*	Isolate Ibaraki-1	Roots	10, 20, 25, 30, 35 and 40 dpi	Expression analysis of nitrilase genes (*BrNIT2*) suggested that 1.1 kb transcripts might be involved in auxin overproduction during clubroot development	[237]
*B*. *rapa* ssp. *Pekinensis*	Isolate Ibaraki-1	Roots	10, 15, 20, 30, 35 and 40 dpi	Expression of AO gene, *BrAO1* increased with clubroot development	[238]
*B*. *rapa* ssp. *pekinensis*	Isolate Ibaraki-1	Roots	10, 15, 20, 23, 27, 30, 35, 40 and 60 dpi	*P*. *Brassicae* infection transiently stimulates the transcription of *BrIPT1*, *3*, *5*, and *7* (cytokinin synthase genes) before club formation	[239]
*B. rapa*	Race 4	Roots	0, 3, 9 and 20 dai	Plant hormone signal transduction, plant-pathogen interaction, and fifteen hub genes (*RIN4* and *IAA16*) were involved in immune response	[240]
*B. napus*	Pathotype 4	Total root tissue	20 dai	The pyramided line (618R) strongly triggers multiple resistance pathways	[241]
*B*. *napus* subsp. *rapifera* Metzg	Pathotype 3A	Total root tissue	7, 14, and 21 dai	In the resistant cultivar, genes related to calcium signaling and genes encoding LRR receptor kinases, RBOH, WRKYs, erfs, and basic leucine zippers were up-regulated	[242]
*B. napus*	Pathotype 4	Roots	12, 24, 60, and 96 hpi	Genes associated with plant hormone signal transduction, fatty acid metabolism, and glucosinolate biosynthesis were involved in regulation of clubroot resistance	[243]
*B*. *napus*	Pathotype 3	Roots	10 dai	The gene *CRF4*, a component of cytokinin signaling pathway play a fundamental role in clubroot resistance.	[203]
*B. napus*	Field isolates (Fuling, China) and pathotype 4	Total root tissue	0, 3, 6, 9, and 12 dai	Host intercellular G proteins got activated together with the enhanced Ca^2+^ signaling, promoted ROS production and PCD in the host plant.	[191]
*B*. *napus*	Field isolate (Kunming, China)	Roots	20 dai	High clubroot resistance was due to the induced expression of broad-spectrum and clubroot specific (*Crr1* and *Cra*) resistance genes	[244]
*B*. *napus*	Pathotype 5X	Roots	7, 14 and 21 (dai)	Immune related genes are associated with SA-mediated responses. JA-mediated responses were inhibited in the resistant genotype.	[245]
*B*. *napus*	Pathotypes 5I (P5I) and 5X (P5X)	Total root tissue	7, 14, and 21 dai	13 genes encoded high cysteine content proteins and three genes encoded proteins with an RXLR motif	[246]
*B*. *napus*	Pathotype 3	Roots and leaves	5, 7, 10, 14 dpi	Up-regulation of phenylpropanoid pathway genes were involved in lignin and flavonoid biosynthesis	[247]
*B*. *napus*	Pathotype 4	Roots and leaves	3, 7, and 10 dai	*BnAAO4* might be directly responsible for overproduction of IAA during early infection	[192]
*B*. *napus* subs. *napus*	Pathotype 6	Roots, leaves, galls	2, 5, 7, 10, 15, 22, 35, 42, and 49 dai	In the resistant roots, higher basal level of SA was stimulated via *ICS1* expression earlier than the susceptible cultivar	[181]
*B*. *napus*	Pathotype 4	Roots	3, 7, 14, and 28 dai	Expression of *BnMYB28.1* was significantly enhanced following treatment with exogenous JA	[223]
*B. napus*	17/31/31 (ECD)	Roots	-	21 genes and 82 candidate genes potentially involved in clubroot resistance were identified	[248]
*B*. *juncea* var. *tumida* Tsen	Field isolate (Chongqing, China)	Total root tissue	15dai	Resistance related genes were involved in PRRs, PTI and ETI signaling pathways, calcium influx, salicylic acid pathway, reactive oxygen intermediates, MAPK-cascades, and cell wall modification	[249]
*B*. *juncea* var. *tumida* Tsen	Field isolate (Fuling, China)	Roots	10, 15, 20, 25, 30 and 40 dpi	Six resistance-related genes encoding ethylene responsive TF, abscisic acid receptor, CDPK-5, quinone reductase gene, MYB family TF and a heat shock TF were up-regulated	[250]
*B*. *campestris* ssp. *chinensis* Makino	Race 7	Roots and leaves	40 DAG	Expression levels of genes encoding *SOD*, *APX*, *CAT*, and *GR* were enhanced	[251]

dai, days after inoculation; dpi, days post inoculation; hpi, hours post inoculation; DAG, days after germination.

### 4.2. Cell Wall Modification and Lignification

**Figure 2:**
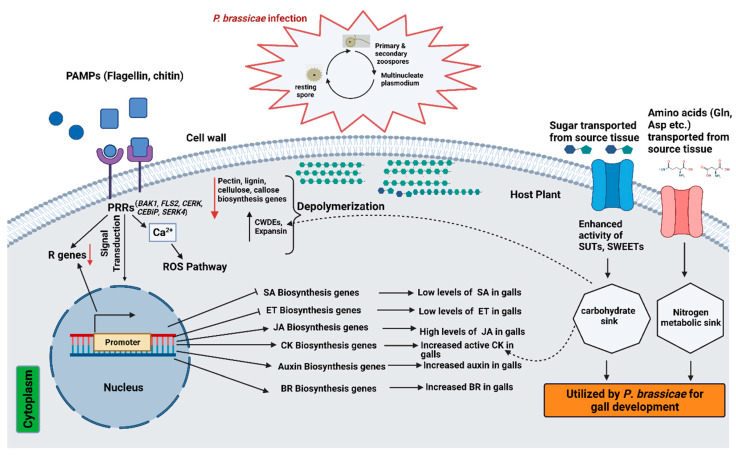
Schematic view of a model depicting metabolic and transcriptional changes happening in susceptible host after *P*. *brassicae* infection adopted and modified from Hasan et al. [263] with copy right permission (License Number—1236246-1) from Canadian Science Publishing and Copyright Clearance Center (www.copyright.com, Accessed on 16 June 2022).

Plant cell walls are very dynamic, act as a physical barrier against pathogen invasion, and can be remodelled by the plants or pathogens [252]. Plant-microbe interactions can cause drastic changes in the chemical composition of the cell wall, including cellulose, hemicellulose, pectin, lignin, callose etc., as a consequence of infection. During *P*. *brassicae* infection, a series of physiological changes occur, including the formation of characteristics root gall, leading to cell wall modification and reorganisation, which differs significantly from the symptomless roots. While pectin plays an important role in cell expansion, adhesion, strength, and porosity, lignification protects cell wall polysaccharides from degradation [253]. Down-regulation of genes involved in the biosynthesis of cellulose, hemicellulose, pectin, callose, and lignin decreased root cell wall rigidity and stability of the clubroot infected cells of Brassica plants [254]. On the contrary, enzymes responsible for degrading these elements were up-regulated in clubroots. Enzymes such as polygalacturonases, pectate lyases, pectin methylesterases (PMEs), and pectin acetylesterases degrade pectin, whereas glycosyl hydrolases family 9 degrades cell wall. Xyloglucan endotransglucosylases/hydrolases or expansins and expansin-like genes are involved in cell wall loosening and elongation processes. PMEs, which regulate the permeability and stability of cell walls, were up-regulated in *B*. *oleracea* during *P*. *brassicae* invasion [184]. Most of the cell division and expansion-related genes were down-regulated in the clubroot-resistant NIL of *B*. *rapa* [185]. However, to protect the plant cells from excessive degradation from pectinase, host plants produce enzyme-inhibiting enzymes, such as PMEIs (PME-inhibitor) and PGIPs (PG-inhibiting protein). In the clubroots of Brassica plants, the genes coding these inhibitors were down-regulated [254]. Plant cell-wall-degrading enzymes (CWDEs) play an important role in loosening and the breakdown of cell wall components [255] and establishment of pathogens [256]. In a study, genes involved in cellulose [cellulose synthase and cellulose synthase-like D], hemicellulose [fucosyltransferase and cellulose synthase-like A], and pectin (*GAUT*) biosynthesis were activated in the resistant genotype at early and secondary infection stage in *B*. *oleracea* [198]. *GAUT* is a key enzyme involved in the biosynthesis of pectic polysaccharide homogalacturonan, which is essential for cell wall formation [257]. Several *GAUT* genes were up-regulated in the clubroot-resistant wild cabbage in response to *P*. *brassicae* infection [183]. At the same time, genes related to β-glucosidase (involved in cellulose hydrolysis) were up-regulated in the susceptible genotype. Lignin synthesized through phenylpropanoid metabolism can be induced as a physical barrier against pathogens [258]. In clubroot-susceptible Arabidopsis, three genes, *4-coumarate-CoA ligase* (4CL1), *cinnamyl-coenzyme A reductase* (CCR1), and *cinnamyl alcohol dehydrogenase* (CAD5), involved in lignin biosynthesis were reported to be down-regulated [259,260]. In contrast, these lignin biosynthesis genes were activated at the early infection stage in *A*. *thaliana*, causing accumulation of lignin except CAD5 [195]. Similarly, in *B*. *oleracea*, genes encoding caffeoyl-CoA-O-methyltransferase (CCoAOMT), CCR1, and peroxidase were up-regulated in the resistant genotype, suggesting a vital role played by lignin in host defence against *P*. *brassicae* [198]. *PAL* (L-phenylalanine ammonia-lyase) is the entry point enzyme of the phenylpropanoid pathway induced by the pathogens [261]. In *B*. *rapa*, up-regulation of the *BrPAL1* gene has indicated that basal defence response induction by clubroot resistance gene *Rcr1* was activated via the phenylpropanoid pathway [236]. Conversely, *PAL* and other genes involved in lignin biosynthesis (*C4H, 4CL, COMT, CCR1, F5H*) were down-regulated in clubroot infected tissue of *B*. *oleracea* [254]. Guaiacyl and syringyl are the vital components of the cell wall of angiosperms [262], and six genes associated with peroxidase are involved in the biosynthesis of guaiacyl, and syringyl lignin were up-regulated in clubroot-resistant wild cabbage [183]. Hence, it is obvious that *P*. *brassicae* infection reduces the rigidity of the root cell wall of the host plant by down-regulating the composition of cell wall and up-regulating CWDEs (Figure 2). In contrast, pectin deposition and lignification processes could play critical roles in host defence against *P*. *brassicae* infection.

### 4.3. Role of Sugars and SWEET Genes in Clubroot Disease Response

*P*. *brassicae* is dependent on the host cell for its nutrient during gall formation. During disease progression, the development of galls on the root system is a consequence of the translocation of carbohydrates from shoots to roots, constituting a metabolic sink. Sugars are the primary energy sources of hosts and pathogens and are transported via monosaccharide transporters, sucrose transporters, and sugars will eventually be exported transporters (SWEETs) [264,265,266,267] (Figure 2). The biotrophic pathogens extract sugars from the host cells [268,269] for their growth and development. In *P*. *brassicae*, 2% of sucrose solution promotes resting spore germination [270], and in the galls of *P*. *brassicae*-infected *A. thaliana* and Brassica plants [271,272], soluble sugars are accumulated [273,274]. In addition, trehalose also gets accumulated in the galls during clubroot development [274]. Hence, galls could act as a carbon sink in clubroot development. Transcriptome studies in *A*. *thaliana* revealed the up-regulation of several genes involved in sugar transport and metabolism during gall formation [179,275]. Apart from sucrose, glucose and fructose transport is regulated via sugar transporter proteins (STPs) and hexose transporters (HTs) [276,277]. Recently, 22 *BoSTP* genes with a conserved sugar transporter domain have been identified in *B*. *oleracea* [230]. The expression of two STP genes, *BoSTP4b* and *BoSTP12*, were induced in clubroot-susceptible cabbage indicating its involvement in monosaccharide unloading and carbon partitioning during galls development. Irani et al. [278] reported the differential expression of genes involved in sucrose and starch biosynthesis upon *P*. *brassicae* attack in *A*. *thaliana*. A significant increase in glucose and fructose in the roots of clubroot-susceptible plants was observed compared to the resistant plants of *A*. *thaliana* [279]. Pathogens compete with the host plants in utilizing the sugars secreted by the host plants for growth and development to sustain their life cycle [280,281]. The competition for sugars is controlled by the sugar transporters stated above [266,280], and regulation of these transporters will throw light on the disease control mechanisms in *Brassica* spp. The *SWEET* genes are responsible for sugar transport across cell membranes and are involved in diverse physiological processes in different plant species [282]. *SWEET* genes act as susceptible genes, and the recessive alleles of *SWEET* genes provide resistance [283]. *SWEET* proteins play a critical role in host-pathogen interactions and are often targets of extracellular pathogens [282,284,285], regulating carbon transport during parasitism and pathogen interaction. Pathogen infection modulates the expression profiles of *SWEET* genes in obtaining sugar during host-pathogen interaction. In *B*. *rapa*, several *BrSWEETs* homologs were up-regulated significantly in clubroot susceptible -NILs compared to the control plant [282]. In addition, slower gall formation in the sweet11 mutant of Arabidopsis compared to the wild-type plants confirmed the *SWEET* gene’s role in clubroot disease development. Hence, it is apparent that *P*. *brassicae* colonization may trigger active translocation and sugar partitioning between the source and the clubbed root tissues, thereby activating the genes involved in sugar transport and metabolism.

## 5. ncRNAomics

Non-coding RNAs (ncRNAs) have housekeeping or regulatory roles [286]. ncRNAs, such as long non-coding RNAs (lnc RNAs) and micro RNAs (miRNAs), play essential roles in response to biotic and abiotic stresses in plants [287,288]. miRNAs are small non-coding RNAs, highly conserved, and regulate the gene expression by post-transcriptional repression [289,290]. miRNAs bind to the target mRNA’s 3′ untranslated region (UTR) to control their expression [291]. Plant miRNAs provide resistance against invading pathogens by interacting with several regions of R genes. Although, plant miRNAs have demonstrated their role in immune response against viruses, bacteria, and fungi [291,292], relatively few studies have exhibited their functions in host defence response against *P*. *brassicae* infection in *Brassica* spp. (Table 4). Verma et al. [293] characterized the changes in miRNA expression profiles of canola roots and identified *P*. *brassicae*-responsive putative miRNAs regulating the genes involved in clubroot disease initiation and progression, elucidating post-transcriptional regulation of the target genes during pathogenesis. Genome-wide identification of miRNAs and their targets in *B*. *rapa* reported that miR164a decreased with *P. brassicae* stress [294]. miR164, reportedly involved in auxin homeostasis [295], was hypothesized to be related to clubroot development. Paul et al. [296] revealed the role of a mi-RNA (Bra-miR1885b) in cleaving the resistance gene Bra019412, confirming that miR1885 is critical in regulating the TIR-NBS gene expression during host defence against *P*. *brassicae* infection. In a comprehensive study, Li et al. [297] identified six antagonistic miRNA-target pairs involved in root development, hypersensitive cell death, and chloroplast metabolic synthesis pathways. The elucidation of cross-talk between miRNAs and their targets may throw new light on understanding the molecular mechanisms of resistance against *P*. *brassicae*.

Long noncoding RNAs (lncRNAs) are important regulatory molecules mediating gene modulation in response to various biotic and environmental stresses in plants and are poorly conserved. lncRNAs exert their regulatory effect through DNA methylation, acetylation, architectural modifications of DNA, and RNA-RNA interactions [298,299]. lncRNAs also regulate the protein-coding genes through cis- or trans-acting processes [300]. A lncRNA-mRNA regulatory network identified the genes regulated by lncRNAs, and 2344 interactions were detected between 1725 mRNAs and 103 lncRNAs [301]. A comparative analysis of the lncRNAs with the clubroot resistance QTL intervals revealed eight lncRNAs localized near the clubroot-resistant QTLs (*Anju1*, *Rcr8*, *CRd*, *CRs, qBrCR38-1*, and *qBrCR38-2*). Through the strand-specific lncRNA-Seq approach, 530 differentially expressed (DE) lncRNAs were identified, with 24 in ch. A8 [302] known to carry QTLs introgressed from the rutabaga which confer resistance against five pathotypes of *P*. *brassicae* [303]. Out of 24, eight lncRNAs were expressed in the resistant plants, and the genes targeted by these lncRNAs were involved in the plant-pathogen interactions, including defensin, pathogen-related protein, and disease resistance protein (NBS-LRR). The same research group identified 464 DE lncRNAs associated with clubroot resistance in *B*. *napus* carrying resistance against pathotype 3 on ch. A3 [304]. A comparative analysis of these lncRNAs with the previously reported DE lncRNAs [302] found 10 lncRNAs regulating defence-related genes. Hence, lncRNAs might play a fundamental role in conferring clubroot resistance through their involvement in transcriptional and post-transcriptional regulation of the defence responsive genes. However, understanding the biological functions of these ncRNAs and their target genes need further research.

**Table 4:** Published ncRNA studies in *Brassica* spp. on clubroot resistance.

Species	Target Tissue	Pathotype /Race/Isolate	Time Point (Tissue Collection)	Inference	Reference
*B*. *napus*	Total roots	Pathotype 4/Pb1	20 dpi	Six antagonistic miRNA-target pairs associated with root development, hypersensitive cell death, and chloroplast metabolic synthesis were identified in the clubroot resistant line	[297]
*B. rapa* ssp. *pekinensis*	Roots	Race 4	15 dai	The putative target genes of the miRNAs were involved in seleno compound metabolism and plant hormone signal transduction	[294]
*B*. *napus*	Roots	Pathotype SACAN03-1	10 and 20 dpi	Several target genes TF, hormone-related genes, genes associated with cytokinin, auxin/ethylene response elements were identified	[293]
*B. rapa*	Leaves and roots	Race 4, Uiryeong, and Banglim	1.5, 3, 6, 12, 24, 48, 72, 96 hpi, and 15 dpi	Cleavage of Bra019412 by Bra-miR1885b suggested that miR1885a negatively regulate the TIR-NBS gene expression during clubroot infection in *B*. *rapa*	[296]
*B. napus*	Roots	Pathotype 3	6, 10, 14, 18 and 22 dpi	24 DE lncRNAs were identified on chromosome A8 known to carrying QTLs conferring resistance against five pathotypes of *P*. *brassicae*	[302]
*B*. *napus*	Roots	Pathotype 3	0 hpi and 10, 14, and 22 dpi	Target genes regulated by DE lncRNAs belong to plant-pathogen interaction, hormone signalling and primary and secondary metabolic pathways	[304]
*Brassica campestris* ssp. *chinensis Makino*	Roots	Race 7	6 wpi	15 mRNAs involved in lncRNA-mRNA co-expression network belong to defense response proteins, protein phosphorylation, root-hair cell differentiation, SA biosynthetic regulation process	[301]

dai, days after inoculation; dpi, days post inoculation; wpi, weeks post inoculation.

## 6. Proteomics

Proteomics deals with the high-throughput analysis of protein’s structure, function, localization, protein-protein interactions, and role in stress response [305]. Proteins are regarded as an essential tool in functional genomics to analyze major signaling and biochemical pathways involved in plants’ responses to environmental stimuli. Unlike the genome, the proteome is highly dynamic and tends to change based on temporal or environmental factors. Proteomic studies focusing on post-translational modifications, subcellular localization and compartmentalization, signaling pathways, and protein-protein interactions can open up new methods for crop improvement [306], which is discussed later. Protein translates the genomic information into functional information and plays a key role in understanding the molecular mechanisms of plant-pathogen interactions [307,308].

Several techniques have been used to study proteomics. The most widely used methods for identifying and characterizing the separated proteins are gel-base two-dimensional polyacrylamide gel electrophoresis (2DPAGE or 2-DE) and the gel-free shotgun proteomics approach [309,310]. 2-DE was the most popular technique for protein separation, which resolves the proteins based on isoelectric point and molecular mass [311]. Later, technological advances have allowed the targeted mass spectrometry (MS) based quantitative approach to be a powerful technique for proteome analysis. The fourth-generation MS techniques, including selective reaction monitoring (SRM), multiple reaction monitoring (MRM), and parallel monitoring reaction (PRM) [312,313], do allow a comprehensive understanding of differentially expressed proteins contributing to abiotic and biotic stress responses. Among several modern proteomics techniques, iTRAQ-based quantitative proteomics has gained popularity for the high-throughput profiling of proteins. Recently, X-ray crystallography and NMR spectroscopy have been developed to determine the three-dimensional structure of proteins, which would help decipher the biological functions of proteins [314]. In past decades, several proteomic studies were framed to elucidate the dynamic changes in protein composition related to metabolic and signaling pathways involved in host defence against *P*. *brassicae* (Table 5). 

A proteomic analysis suggested that *P*. *brassicae* infection metabolizes the fatty acids from the host causing fatty-acid degradation as the infection progresses, indicating the participation of fatty-acid signaling in *P*. *brassicae* infection [315]. Differentially expressed proteins (DEPs) analysis revealed that proteins involved in the glutathione transferase activity pathway could reduce the damage caused by *P*. *brassicae* by catalyzing glutathione and electrophilic compound [316]. A label-free shotgun proteomics was followed to gain further insights into clubroot regulations at the post-transcriptional level in *B*. *rapa*. Based on functional annotations of differentially accumulated proteins (DAPs), a novel signaling pathway acting in a calcium-independent manner via a cascade of MAPK contributing to clubroot resistance (mediated by the resistance gene *Rcr1*) was hypothesized [317]. Another proteomic study in Chinese cabbage demonstrated the up-regulation of cycloartenol synthase (CAS1) and cytochrome P450 51G1 proteins involved in the synthesis of brassinosteroid (BR) at the secondary stage of *P*. *brassicae* infection [315]. The role of CAS1 in clubroot development was confirmed by treating the clubroot-infected plants with an inhibitor of CAS1, resulting in reduced root gall size. Based on the proteomic studies, it became apparent that in addition to auxin, cytokinin, and JA, brassinosteroids and fatty-acid signaling pathway also regulate clubroot disease development.

**Table 5:** Published proteomic analyses in *Brassica* spp. during interaction with clubroot.

Species	Target Tissue	Pathotype/Race/Isolate	Time Point (Tissue Collection)	Methodology	Inference	Reference
*B*. *oleracea*	Leaves	Field isolate (Gangneung, Korea)	5 dai	2-DGE, MALDI-TOF/TOF MS	The resistant plants showed an increased abundance of ABA-responsive protein, fructose-bisphosphate aldolase and glucose sensor interaction protein, mediating basal defence against *P*. *brassicae*	[318]
*B*. *oleracea*	Roots	-	4 wai	2-DE	cytokinin may not cause the tumorous growth and the protist was inhibiting host gene expression causing host protein degradation leading to gall formation	[319]
*B*. *rapa* ssp. *pekinensis*	Roots	Race 4	70 dai	2-DE, MALDI-TOF	10 DEPs responded to stimulation of which two were involved in SA signaling pathway.	[320]
*B*. *rapa*	Roots	Pathotype 3	15 dpi	UHPLC-MS/MS	Functional annotation of 527 DAPs suggested a novel signaling pathway acting in a calcium-independent manner through an unique MAPK cascade	[317]
*B*. *rapa* ssp. *Pekinensis*	Roots	Field isolate (SAU, China)	5 dai	2-DE, MALDI-TOF/TOF MS	Proteins related to SA-mediated SAR and JA/ET-mediated ISR were identified showing some degree of cross-talk	[213]
*B*. *rapa*	Roots	Race 4	3 dai	2-DE, LC/MS/MS	Resistant line produced more ATP-binding protein for the ABC transporter whereas the susceptible line exhibited increased levels of PR1 production	[321]
*B*. *rapa*	Roots	Field isolate (Songming, Kunming, China)	14, 21, 28, 35, and 42 dai	iTRAQ, HPLC-MS/MS	DEPs were associated with the glutathione transferase activity pathway and significantly enriched cytokinin signaling or arginine biosynthesis pathways, both of which were related to stimuli and plant defense reaction	[316]
*B*. *rapa* subsp. *pekinensis*	Roots	Isolate Pb2	0, 10 and 20 dai	2-DE, iTRAQ, LC-ESI-MS/MS	Proteins involved in brassinosteroids metabolism (CAS1, CYP51G1) were up-regulated after inoculation	[315]
*B*. *napus*	Roots	Pathotype 3	12, 24, 48, and 72 hai	2-DE, LC/MS/MS	Reduction of adenosine kinase indicated the role of cytokinin in clubroot infection and decreased intensity of CCoAOMT abundance suggested a reduction in host lignin biosynthesis upon pathogen attack	[199]
*B. napus*	Roots	Pathotype 3	7, 14, and 21-DPI	LC-MS/MS	73 putative proteins orthologous to clubroot-resistant proteins and QTL associated with eight CR loci in different chromosomes including A3 and A8 were identified	[322]

dai, days after inoculation; dpi, days post inoculation; wai, weeks after infection; hai, hours after inoculation.

## 7. Metabolomics

Metabolomics refers to the comprehensive study of metabolites participating in various cellular events in a biological system [323]. Metabolomics is placed at the phenotypic end of the -omics spectrum capturing the information starting with the genome and progressing through transcriptome and proteome [324]. Qualitative and quantitative metabolites profiling of different tissues, cells, and organs can help identify and quantify primary and secondary/specialized metabolites of plants used in critical biological processes. Metabolomics helps analyze the changes in host plant metabolism in response to pathogen attack, thereby providing a better understanding of host-pathogen interactions. The activation/deactivation of metabolites associated with different signaling pathways determines the resistance or susceptible outcome of a plant-disease interaction [325]. In recent years, multiple separation and analytical techniques such as MS, liquid, and gas chromatography-MS (LC-MS, GC-MS), Fourier-transform ion cyclotron resonance mass spectrometry (FT-ICR-MS), capillary electrophoresis liquid-chromatography mass spectrometry (CE-MS), and NMR have been developed to analyze the plant metabolites (reviewed by Raza et al. [326]). In plants, metabolomic analyses can be accomplished by both non-targeted [327] and targeted [328] approaches. While untargeted metabolomics deals with both secondary and primary metabolites, the targeted approach identifies glucosinolates (GSLs). Over the years, several metabolomic studies in different Brassica crops evaluated the effects of phytohormones, secondary metabolites and GSLs to evaluate conferring resistance to *P*. *brassicae* (Table 6). 

During gall formation, a plethora of changes occurs in the primary and secondary metabolism (Reviewed by Ludwig-Müller et al. [22]). Primary metabolism is involved in a plant’s growth, development, and reproduction and revolves around critical physiological compounds such as sugars and amino acids. In the previous section, we have discussed about the role of carbohydrates in constituting a metabolic sink during the development of galls in the root system. In contrast to carbohydrate metabolism, little information about nitrogen metabolism during clubroot disease development is available, except for a few studies [329]. A study on cabbage has shown that total nitrogen, protein, and amino-acid levels increased in the galls of cabbage during clubroot development [271]. A study has suggested that clubroot susceptibility was positively correlated with the accumulation of several amino acids at the early infection stage in *B*. *napus* [330]. In clubroot infected Arabidopsis, a large amount of proline accumulates, which could be attributed to the response to water limitation caused by the loss of functional roots [182]. The induction of arginine constitutes the basal defence mechanism by reducing hormone-triggered cellular proliferation during gall development [222]. A difference in arginine metabolism signature was reported in susceptible and partially resistant Arabidopsis plants. Specifically, in susceptible plants, a massive induction of arginine was found in the later stage of infection [182].

During gall formation, pathogen alters the levels of plant growth hormones, especially auxins and cytokinins, important for hypertrophy of the infected roots. In *B*. *rapa*, compared to resistant genotype, the susceptible genotype showed higher auxin and cytokinin levels after *P*. *brassicae* infection except trans-zeatin and 3-indolebutyric acid [331]. In *A*. *thaliana* and *B*. *rapa*, IAA levels increased at different phases of clubroot infection [225,332,333,334]. IAA biosynthesis in Brassicaceae proceeds via indole GSLs through the formation of indole-3-acetaldoxime (IAOX), indole-3-methyl glucosinolate (IMG), and indole-3-acetonitrile (IAN). During the invasion of the pathogen, IMG converts to IAN by myrosinase, thereby increasing the levels of auxin precursor. Studies have shown that in clubroot-infected *B*. *rapa* roots, IAN is specifically converted to IAA from indole GSLs [335]. The enzyme nitrilase may regulate IAA biosynthesis in Brassicaceae by converting IAN to IAA, and nitrilase activity with increased IAA content during root gall development has been reported in Chinese cabbage [336,337]. Grsic et al. [225] reported an enhanced activity of nitrilase and myrosinase at the late stage of clubroot development in Chinese cabbage. Hydrolysis of free IAA from inactive auxin conjugates could be another pathway for IAA biosynthesis. A study in *B*. *rapa* reported hydrolysis of several IAA-amino acid conjugates during clubroot infection [333].

In addition to auxin, increased concentrations of free and bound cytokinins have been reported during clubroot disease development. *P*. *brassicae* can form cytokinins in a limited amount by converting trans-zeatin and its riboside [338]. Devos et al. [339] reported a greater amount of active cytokinins such as zeatin early in the infected *B*. *rapa* plants. Though *P*. *brassicae* requires cytokinin for its development, it can synthesize only small amounts of cytokinin [180], prompting it to take cytokinins from its host. In the host, cytokinins help develop vascular cambium essential for normal plant growth and act as a route for *P*. *brassicae* to hijack host development for gall formation. Malinowski et al. [180] reported that gall formation is associated with a decline in cytokinin content, and other phytohormones may be involved in the hypertrophy of clubbed roots. The down-regulation of cytokinin can be a part of the plant-defence mechanism against *P*. *brassicae*. It reduces the signals involved in cell division and the cell cycle progression in the root cortex [200]. Disturbing the balance of cytokinin synthesis and degradation may impact clubroot disease progression.

The key hormone involved in the defence response against biotrophic pathogens is SA, and the application of exogenous SA was reported to curb clubroot disease in Arabidopsis [259]. SA level was enhanced in the partially resistant Bur-0 ecotype of *A*. *thaliana* after *P*. *brassicae* infection with mild elevation of JA [215], indicating that both SA- and JA-triggered defences result in *P*. *brassicae* resistance. But SA appears to be more efficient than JA in conferring resistance. Exogenous SA decreased the clubroot symptoms in two accessions of Arabidopsis [215] and pakchoi [251]. Upon *P*. *brassicae* infection, SA content was increased at later stages of infection in the susceptible cultivar of *B*. *napus* but at an earlier time point in the resistant cultivar [181]. However, mechanisms governing SA to repress clubroot resistance are not very clear. In Arabidopsis, SA and its analogs treatment have reduced auxin level and signaling, essential for gall formation [340]. Xi et al. [251] hypothesized that exogenous SA treatment might influence auxin biosynthesis and signaling, thereby reducing the infection abilities of *P*. *brassicae* and enhancing resistance. Double SA application (before infection and 15 dai) diminished gall formation in the resistant cultivar (Alister) of *B*. *napus* [181]. Interestingly, the latest study has demonstrated a lower accumulation of SA in the resistant line of *B*. *rapa* than that of the control [240]. This could have happened due to the down-regulation of NPR1 homologs (the key regulators of SA-mediated resistance) in the resistant line leading to a significant decrease in SA content.

**Figure 3:**
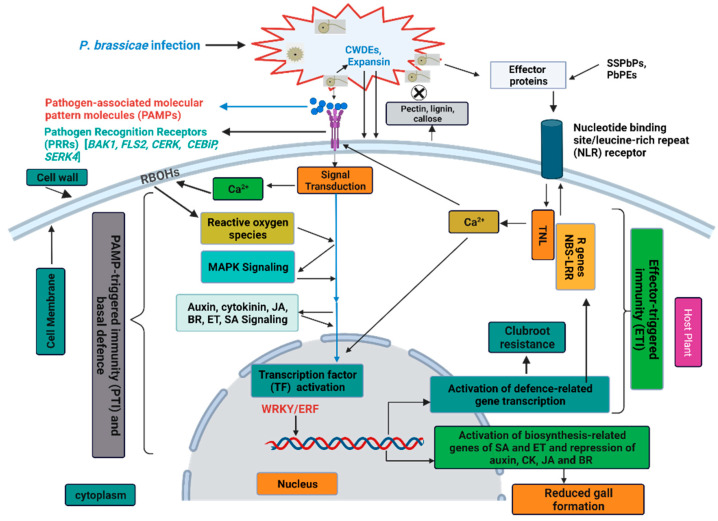
Proposed mechanism of clubroot resistance in Brassica crops.

In contrast to SA, JA plays a vital role in clubroot disease development (Figure 2 and Figure 3) at the secondary infection stage/cortex infection stage. Xu et al. [223] reported a greater accumulation of JA at 14–28 dai in susceptible *B*. *napus* during clubroot infection. Two to three-fold higher JA and its expression level was reported in susceptible Col-0 at the secondary infection stage in Arabidopsis [215]. In a recent study, JA precursor (cis-OPDA), JA-bound (jasmonoyl-L-isoleucine, JA-Ile), and free JA content decreased at 9 and 20 dai in the resistant line of *B*. *rapa* (BrT24), though increased significantly in the susceptible line [240]. Elevation of JA in the susceptible cultivars raises a question of whether this is a part of pathogen strategy to overcome plant defence by suppressing the SA pathway [181,240]. Interestingly, JA also mediates resistance against clubroot at the secondary infection stage, although it is weak. Exogenous JA treatment reduced clubroot symptoms only in the susceptible Arabidopsis (Col-0), indicating its participation in the weak defence against *P*. *brassicae* [215]. Apart from SA and JA, the role of ethylene mediating resistance to clubroot has been demonstrated in *A*. *thaliana* [341,342]. Wei et al. [240] opined that ethylene signaling is required to restrict gall growth. Knaust and Ludwig-Müller [341] have shown that ET response 1 (etr1) and ET insensitive2 (ein2) mutants were more susceptible than the wild-type Arabidopsis. This was in agreement with Siemens et al. [179], who reported the down-regulation of ethylene biosynthesis genes in clubroot-infected *A*. *thaliana*. Devos et al. [339] reported an increase in the content of *1*-*Aminocyclopropane 1-carboxylic acid (ACC)* (direct precursor of ethylene) in the unaffected roots of *B*. *rapa* compared to the infected plants at 14 dpi. But Lan et al. [331] could not observe any change in the levels of *ACC* which could be attributed to different genetic backgrounds of the genotypes. Another phytohormone, brassinosteroids (BR), reportedly mediated clubroot resistance by its inhibition in the resistant line of *B*. *rapa* but was induced in the susceptible line (Figure 3).

Glucosinolates (GSLs) are one of the largest known groups of secondary metabolites in the family Brassicaceae [343]. Based on their amino acid precursors, GSLs can be divided into three groups, aliphatic, aromatic, and indolic GSL [344]. The GSLs have long been associated with clubroot disease, and their role in clubroot disease development has been reviewed extensively by Ludwig- Müller [345]. While aromatic GSLs act as a plant defence against pests, aliphatic GSLs are considered defence compounds against plant pathogens [345,346]. Compared to aromatic and aliphatic GSLs, most studies have focused on indolic GSLs in clubroot disease development. Indolic GSLs are thought to be the precursors of IAA/auxin biosynthesis in Brassicaceae, and high IAA levels are involved in gall formation. The earlier induction of indole GSLs may be responsible for the overproduction of IAA during clubroot disease development. Several studies in the Brassicaceae family proved that indole GSLs content is correlated with clubroot disease development and severity, directly or indirectly [224,332,334,345]. Nonetheless, indolic GSLs were involved in innate immunity response in *A*. *thaliana* [347]. Correlations between low indole GSLs content and clubroot resistance have been observed in the members of Brassicaceae [223,348,349,350]. The elevation of indolic GSLs happened largely due to the accumulation of 4-methoxyglucobrassicin [223,232] and neoglucobrassicin, glucobrassicin [232]. Zamani-Noor et al. [351] reported higher mean contents of 4-methoxyglucobrassicin in *P*. *brassicae*-infected resistant and susceptible plants than non-inoculated plants irrespective of the pathotypes used. So, further study on the function of 4-methoxyglucobrassicin will throw light on the role of indolic GSLs in clubroot disease development. In contrast to indole GSLs, knowledge of aliphatic and aromatic GSLs in clubroot disease is limited. Though aliphatic GSLs are supposed to be involved in defence response, several studies showed the opposite results. Ludwig- Müller et al. [224] reported increased aliphatic GSLs in two susceptible Chinese cabbage varieties but observed no change in the resistant varieties. Xu et al. [223] also reported that aliphatic GSLs were involved in clubroot development in *B*. *napus*. Various components of aliphatic GSLs (such as glucoalyssin) were increased together with the decreased glucoraphanin content in the resistant plants. This summarizes that instead of total components of GSLs, particular components of GSLs are responsible for clubroot resistance or susceptibility. The role of aromatic GSLs in clubroot disease is not very clear, though few authors have reported its enhanced content both in resistant and susceptible plants. Two resistant varieties of Chinese cabbage showed an increase in aromatic GSLs, indicating their role in host resistance [224]. Zamani-Noor et al. [351] observed lower levels of gluconasturtiin (aromatic GSL) in the roots of susceptible varieties of *B*. *napus* compared to resistant varieties. Xu et al. [223] demonstrated enhanced content of aromatic GSLs in both the resistant and susceptible plants at the secondary infection stage, though its component was completely different.

In summary, GSL metabolites, specifically indole GSLs, are more likely to play an important role in disease development than defence response against *P*. *brassicae*. However, a recent study has raised the possibility of GSL breakdown products acting against *P*. *brassicae*. A recent report has shown that the resistant cultivar could maintain water balance through breakdown products of aromatic GSL in the stomatal aperture during *P*. *brassicae* infection [351]. Nevertheless, it requires further research to understand the role of GSL metabolism in clubroot-disease development and defence in the Brassicaceae family.

**Table 6:** Published metabolomic analyses in *Brassica* spp. involved in resistance against clubroot.

Species	Target Tissue	Pathotype/Race/Isolate	Time Point (Tissue Collection)	Methodology	Inference	Reference
*B*. *oleracea*	Leaves and roots	Isolate yeoncheon	0, 3, 6, 12, 24, 72 hai and 15 and 40 dai	HPLC	Plants with higher levels of neoglucobrassicin, glucobrassicin and methoxyglucobrassicin exhibited disease symptoms with gall formation	[232]
*B*. *napus*	Roots	Pathotype 1	14, 21, 28, 35, and 42 dpi	UPLC-MS/MS, GC-MS	Clubroot susceptibility was positively correlated with clubroot-induced amino acids accumulation	[330]
*B*. *napus*	Leaves and roots	Pathotype 4	3, 7, and 10 dai	RP-HPLC/ESI–MS/MS	IAA acts as a signalling molecule to induce root hair infection during early stage of infection whereas NPA treatment reduced the disease index	[192]
*B*. *napus* subs. *napus*	Leaves, roots, galls	Pathotype 6	2, 5, 7, 10, 15, 22, 35, 42, and 49 dai	HPLC	JA promoted gall formation in both the cultivars, whereas SA suppressed gall formation in the resistant cultivar	[181]
*B*. *napus*	Roots	Pathotype 4	3, 7, 14, and 28 dai	HPLC	JA-induced aromatic GSLs were involved in defence response and JA-induced aliphatic GSLs may be involved in clubroot disease development	[223]
*B*. *napus*	Leaves and roots	Isolate P1 and P1 (+) (pathotype 1)	35 dpi	LC-MS	Single and total aliphatic and indolic GSL contents were significantly lower in the leaves and roots of susceptible cultivars compared to the resistant ones	[351]
*Brassica napus* cv. Westar	Roots	Pathotype 3	3, 4, 5 and 6 wpi	HPLC-DAD, HPLC-ESI-MS	Plants produced a complex blend of phytoalexins and other antimicrobial metabolites as a defence response	[352]
*B*. *campestris* ssp. *chinensis* Makino	Leaves and Roots	Race 7	40 DAG	-	Clubroot incidence rate and disease index were decreased after treatment with 0.6 mM exogenous SA	[251]
*B. campestris* ssp. *pekinensis*	Roots	-	5, 10, 14, 20, 24, 28, 32 and 40 dai	HPLC	Indole and aliphatic was higher in the roots of susceptible varieties whereas aromatic glucosinolates was higher in the roots of resistant varieties	[224]
*Brassica campestris* L. ssp. *pekinensis* cv. Granat	Leaves and Roots	-	14, 21, 28, 35, 42 dai	HPLC	JA level was enhanced during club development and may be involved in the up-regulation of three enzymes required for IAA synthesis	[225]
*B*. *campestris*	Roots	-	5, 10, 12, 13 and 14 dai	GC-MS	Mean of IAA content in the infected plant was 66.5% higher than the non-infected plants	[332]
*B*. *rapa* ssp. *pekinensis*	Roots	Field isolate (Songming, Kunming, Yunnan, China)	14, 21, 28, 35, 42 dpi	LC-ESI-MS/MS,	Metabolites related to amino-acid biosynthesis, fatty-acid biosynthesis and elongation, glutathione and glucosinolate metabolism were highly accumulated in the resistant genotype	[331]
*B, rapa*	Roots	Race 4	0, 3, 9, and 20 dai	UHPLC-MS	Inhibition of IAA, cytokinin, JA, and SA contents may play important roles in regulation of clubroot resistance	[240]

dai, days after inoculation; dpi, days post inoculation; wpi, weeks after inoculation; DAG, days after germination.

## 8. Multi-Omics Opened Up New Avenues for Crop Improvement

The traditional breeding techniques mainly based on phenotypic selection are unable to achieve the needs of food security and sustainability crop productivity [353], as several desirable traits are complex in nature. Engineering these complex traits could help crop improvement by a proper understanding of the flow of information from the gene, RNA, protein and metabolite to these traits. To achieve genetic gains for the desirable traits, several molecular breeding approaches, including MAS, MABC, marker-assisted recurrent selection (MARS), GWAS, genomic selection (GS), NGS-enabled QTL-seq, BSR-seq, and GBS techniques, have been used to speed up the breeding process. Combining all these genomic tools with the conventional breeding techniques, called ‘genomics-assisted breeding’ [354], is harnessed by the breeders to practice precision breeding. Furthermore, the sequencing cost has been reduced several folds with the development of NGS technologies and platforms. As a result, genome-sequence assemblies of hundreds of plants became available to understand the relationship of genomic segments with the phenotype. In addition, genomic techniques are widely used for germplasm enhancement by deciphering the genome architecture and genome variations, thereby improving the genetic stocks. Mutagenomics tools such as Targeted Induced Local Lesions IN Genomes (TILLING) has emerged, which is a modern omics technique that causes high-throughput mutations leading to gene modification in the target traits. All these genomic tools could be applied to enhance clubroot resistance of the germplasm/breeding lines in *Brassica* spp. Specifically, combining genomic selection with the multi-omics analysis could improve the prediction accuracy of the trait performance. The advancement of NGS technologies facilitates scRNA (single-cell RNA) sequencing, which provides the information about unique mutations in the cell [355]. Recently, another transcriptomics approach known as alternative splicing (AS) has been used to produce multiple transcripts in response to abiotic stress conditions [356]. Several powerful quantitative proteomics approaches (discussed earlier) are aiding in the identification of proteins involved in the causative function of important traits, including clubroot resistance. The differentially expressed protein can help identify novel protein markers for crop improvement. Several high-throughput metabolomics techniques have been widely used in different crops in response to biotic/abiotic stresses and to unravel the phenotypic plasticity. Metabolomics integrated with other omics approaches such as genomics, transcriptomics, and proteomics could provide a list of various functions ranging from genome to metabolome, including phenotypic characteristics [357]. In recent years, metabolites have been increasingly used to predict phenotyping properties. In the second decade of the 21st century, CRISPR/Cas mediated genome editing technology is extensively used to edit plant genomes. Genome-editing technique integrated with speed breeding will facilitate the rapid validation of the incorporated gene without in vitro manipulations [306]. Multi-omics platforms integrated with genome-editing tools will help develop precision breeding. Finally, the high-throughput phenomics platforms for rapid and precise phenotypic assessment will maximize productivity and promote sustainable crop production. Hence, full automatization of clubroot disease indexing by digital root imaging systems is a long overdue task which needs to be adopted for rapid pathotyping [358]. Often, the digitalization of root architecture and morphology is carried out by analysing the digital root images using different software, which is extremely necessary in the near future for disease indexing of clubroot disease. The multi-omics technologies are generating huge data, known as ‘big data’, whose integration has been a challenging task. So, deep learning (DL) has emerged as a powerful approach to integrate multi-omics data [359], which can encode and model many forms of complex data. Different omics tools have led to the emergence of different technology platforms, methods and computational tools, and the combination of these tools will provide sufficient power to enhance clubroot resistance in *Brassica* spp.

## 9. Concluding Remarks and Perspectives

**Figure 4:**
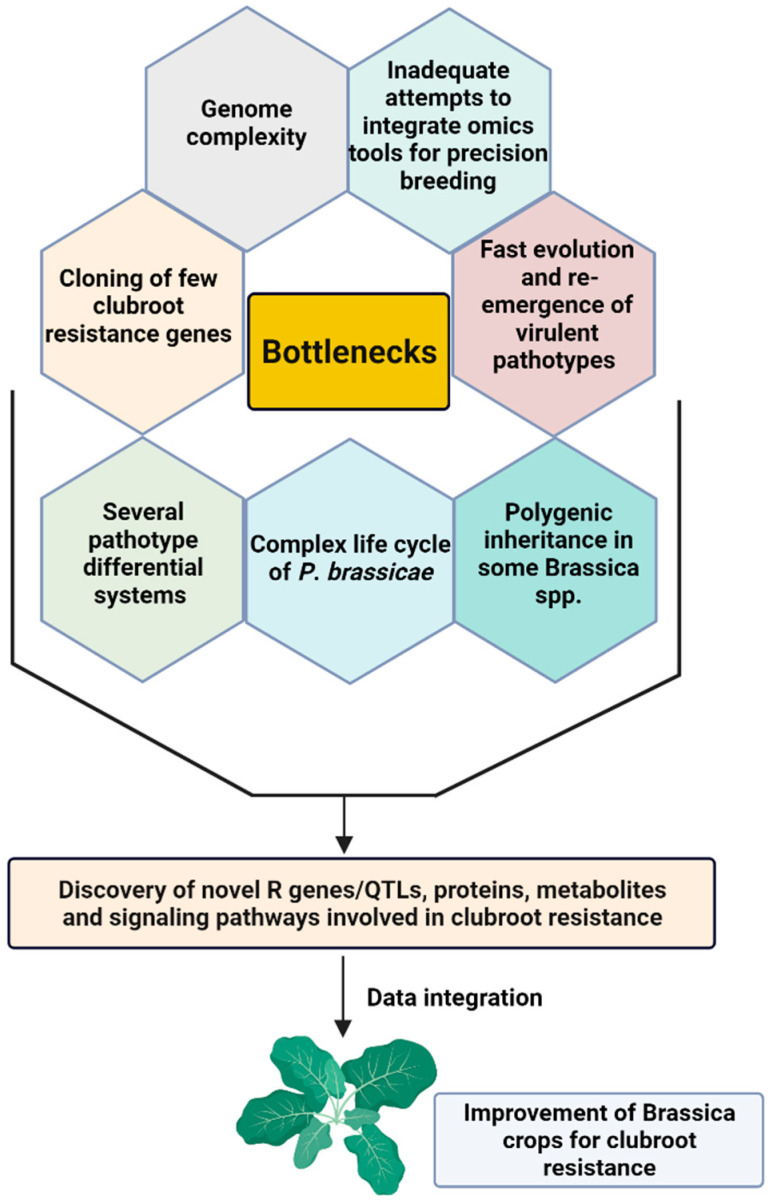
Existing bottlenecks in the utilization of multi-omics approaches to develop clubroot-resistant Brassica crops.

In recent years, the application of omics technologies has provided insights into the molecular mechanism of disease resistance at the genomic, epigenomic, transcriptomic, proteomic, and metabolomic levels. If these technologies are integrated at the right scale, they may decipher the multi-dimensional properties of plant diseases [360]. In this review, we have discussed the recent advances made in various omics techniques to understand the mechanism of clubroot resistance in Brassica crops. Still, several challenges (Figure 4) remain to realize the full potential of omics techniques in managing clubroot disease.

Effective deployment of R genes in clubroot resistance breeding demands detailed knowledge of the ecology and life history of the host, pathogen, and their interactions, allowing the host resistance prediction at different stages of infection. While *B*. *rapa* remains a good source of many race-specific, dominant R genes, *B*. *oleracea* has a complex inheritance pattern and a more continuous resistance involving major and minor genes. This pointed towards different physiological resistance mechanisms imparted by *B*. *rapa* and *B*. *oleracea*, resulting in complete resistance in *B*. *rapa*, which is lacking in *B*. *oleracea* [30,361]. Hence, it becomes difficult to fine map or clone resistance genes, restricting R genes’ utilization in resistance breeding in *B*. *oleracea.* Combining both race-specific and race-nonspecific genes may be an effective and durable strategy to potentially increase resistance against *P*. *brassicae*. In the Brassica-*P*. *brassicae* pathosystem, several R genes have been mapped, but only three have been cloned. Moreover, the molecular mechanism of resistance of these cloned genes has yet to be clarified. Historically, cloning of R genes has been accomplished by map-based cloning. However, in the post-genomics era, significant accomplishments have been made in cloning R genes in several crops including, Brassica that have complex genomes.

To date, in *Brassica* spp. different research groups have identified many clubroot-resistant QTLs. Nevertheless, comparing these QTLs is impossible due to the lack of common molecular markers, use of different clubroot resistant sources, and *P*. *brassicae* pathotypes/isolates. Besides, various researchers have named QTLs/genes independently. However, many of these belong to the same genomic region leading to naming some of these loci by other names. Accurate and unambiguous naming of these loci would help in efficient deployment these genes in breeding of clubroot resistance. Different isolates could be functionally different in their pathogenesis; therefore, it is important to develop isolate/pathotype-specific clubroot-resistant lines, and molecular markers need to be developed to detect isolate-specific resistant lines.

In *Brassica* spp., R gene identification for clubroot disease has focused mainly on NBS-LRR genes. Hence, future studies should analyze the contribution of other classes of R genes, such as RLKs, RLPs, and wall-associated kinases in clubroot resistance, which may facilitate the cloning of novel R genes. In this regard, pangenome study will help identify a large R gene repertoire (RGAs) in *Brassica* spp. Pangenomics is a promising approach for studying the structural variation in the genes and has been proved useful in deciphering the structure, function, and evolutionary origin of R genes. Furthermore, super-pangenome representing the genomes of wild relatives and different species [362] may facilitate the identification of many more novel candidate resistance genes, improving the speed and accuracy of crop breeding by broadening the Brassica gene pool. In addition to the host resistance genes, the investigation of pathogenesis-related genes, effector candidates, transcriptome, miRNA, proteome and metabolome data of *P*. *brassicae*-inoculated tissue is essential to understand the interaction of host-pathogen to formulate the strategy to develop clubroot-resistant Brassica crops (For a comprehensive review, see Jutta Ludwig-Müller [363]).

The genome complexity of *Brassica* spp. has contributed to the complicated gene rearrangement, duplication, and loss [364], narrowing the scope of utilization of R genes in the breeding programs. This has also resulted in the loss of genetic diversity in *B*. *napus* compared to its progenitors, *B*. *rapa* and *B*. *oleracea* [365]. Clustering of R genes is prevalent in clubroot disease, further providing evidence of genome complexity. Clustering of R genes may help recognize diverse pathotypes carrying different *Avr* genes; however, clustering and duplication may be problematic in R gene identification as these genes may collapse in genome sequence assemblies [366]. At times, R-genes from the parents get masked in the resynthesized genomes of the progenies, as occurred in the clubroot R gene [151]. This can be overcome by genome editing techniques such as CRISPR-Cas9, which has been successfully used for the functional gene studies in the Brassica-pathogen system though not performed in the Brassica-*P*. *brassicae* pathosystem.

A huge number of data collected through multi-omics technologies has enabled the construction of network biology, wherein computational and mathematical analysis and modelling predict the pathogenicity and mechanisms of virulence in host-pathogen interactions. While analyzing certain biological processes, integrated multi-omics approaches could complement each other. In this context, systems biology has provided an excellent platform to integrate different -omics technologies to have a holistic understanding of the adaptation and development of an organism [367]. Integration of multi-omics technologies can enhance our understanding of the complete biological systems by predicting the behavior and interactions among genes, proteins, and metabolites with respect to external stimuli [368]. Previously, a comprehensive analysis of multi-omics approaches has increased our understanding of systems biology associated with abiotic stress response in plants [369,370]. However, a model needs to be developed to identify the molecular regulator network involved in clubroot resistance by combining systems biology with multi-omics approaches. With the advancement of computational tools, discoveries involving Brassica-*P*. *brassicae* pathosystem will be made in near future to increase our understanding of the molecular regulator network involved in clubroot resistance in Brassica crops.

## Data Availability

Not applicable.

## Supplementary Materials

The following are available online at https://www.mdpi.com/article/10.3390/ijms23169280/s1. References [371,372,373,374,375,376,377,378,379,380,381,382,383,384,385,386,387,388,389,390,391] are cited in the supplementary materials.
