# Supplementary material for: Multi-Omics Approaches to Improve Clubroot Resistance in Brassica with a Special Focus on Brassica oleracea L
Source: Int J Mol Sci. 2022 Aug 17;23(16):9280. doi: 10.3390/ijms23169280 (PMC9409056; doi:10.3390/ijms23169280)
Supplement: Supplementary file 1 [file ijms-23-09280-s001.zip › ijms-1795035-supplementary.pdf]

Table S1. List of resistance genes/QTLs associated with clubroot diseases in related *Brassica* spp.

Species	Mapping population	Pathotype/Race/Isolate	Gene locus/QTL	Chr/LG	Reference
B. rapa	F2:3, BC1	Race 2	*CRa*	A03	[144]
B. rapa subsp. *pekinensis*	F2, F3	Race 6	*CR6a*	G1	[371]
			CR6b	G3	
B. rapa	F2	Isolates Wakayama-01, Ano-01 (pathotype group 4)	*Crr1*	A08	[145]
			Crr2	A01	
B. rapa	F2	Isolates Wakayama-01 and Ano-01 (pathotype 4)	*Crr4*	A06	[146]
B. rapa	F2:3	Isolate Ano-01	*Crr3*	A03	[372,95]
B. rapa	F2, BC	Race 2	*CRa*	A03	[373]
B. rapa	F2	Isolates M85 and K04	*CRa,*	A03	[147]
			CRk	A03	
			CRc	A02	
B. rapa	F2	Race 4	*Pb-Br3*	A03	[374]
			Pb-Br8	A08	

B. rapa	F2	Race 2	*CRa*	A03	[121]
B. rapa	F2	Race 4, Pathotypes 3	*CRb*	A03	[97,375,96],
B. rapa spp. *chinensis*	BC1	pathotype 3	*RPb1*	A03	[376]
B. rapa	F2	Isolate Ano-01 (pathotype group 4)	*Crr1a, Crr1b*	A08	[120]
B. rapa	BC1F2	pathotypes 2, 4, 7, and 10	*PbBa1.1*	A01	[377]
			PbBa3.1	A03	
			PbBa8.1	A08	
			PbBa*3.2*	A03	
			PbBa3.3	A03	
B. rapa	F2	Pathotype 4	*CRa, CRb,* and CRb^{Kato}	A03	[98]
B. rapa	BC1	Pathotype 3	*Rcr1*	A03	[99]
B. rapa	F_2	Race 4	*QS_B1.1*	A01	[378]
			QS_B3.1	A03	
			QS_B8.1	A08	

B. rapa	F2	Pathotypes 2, 5 and 6	*Rcr1*	A03	[104]
B. rapa	BC1	pathotypes 2, 3, 5, 6, 8	*Rcr4*	A03	[111]
		pathotype 5x	*Rcr8*	A02	
			Rcr9	A08	
B. rapa	F2	Pathotype 3	*Rcr2*	A03	[105]
B. rapa	BC1	-	*CrrA5*	A05	[379]
B. rapa ssp. *pekinensis*	F2	Race 4	*Crd*	A03	[112]
B. rapa	BC1	Canadian field isolates	*BraA.CR.a*, *BraA.CR.c*	A03	[380]
			BraA.CR.b,	A08	
B. rapa	F2	Pathotype 3	*Rcr5*	A03	[108]
B. rapa	F2	pathotype 4	*CRs*	A08	[113]
B. rapa	F2	Pathotype 4	*CRs*	A08	[113]
B. rapa	F2, BC	Pathotype 3	*Rcr6*	A08	[107]
B. rapa	BC1	Pathotypes 3H and 5X	*Rcr3*, $Rcr9^{wa}$	A08	[109]
B. rapa	DH	pathotype "Banglim"	$PbBrA08^{Banglim}$	A08	[114]
	F2	Pathotypes 3H,	*CRa/CRbKato*	A03	[150]

B. rapa subsp. *rapifera*		5X and 5G	*Crr1*	A08	
B. campestris ssp. *Chinensis* Makino	F2	Race 7	*qBrCR38-1*	A07	[381]
			qBrCR38-2	A08	
B. napus	DH	Pb137–522 and K92–16	*Pb-Bn1* (DY4)	DY4	[382]
			Two QTLs	*DY4*, *DY15*	
B. napus	DH	Isolates Korporal, K, 01.07, 1, 01:60, e4x04, and a	Nineteen QTLs	N02, N03, N08, N13, N15, N16 and N19	[151]
B. napus	DH	Pathotype 3	*CRa* or *CRb*	A03	[383]
Brassica napus var. *napobrassica*	DH	Pathotypes 2, 3, 5, 6, and 8	*QTL*	A08	[303]
B. napus	DH	Pathotype 3	*CRa*	A3	[384]
B. napus	Association mapping population (472)	Pathotype 4	*MCR-C3*	C03	[385]
			MCR-C9	C09	
			MCR-A4	A04	
			MCR-C3	C03	
			SCR-A10a	A10	

			SCR-C6	C06	
			SCR-A10b	A10	
			SCR-C3	C03	
			SCR-C4a	C04	
			SCR-C4b	C04	
B. napus	DH	Isolates eH and K92-16.	20 QTLs (Isolate eH)	A05, A07, C01, C02, C03, C09	[386]
			13 QTLs (Isolate K92-16)	A03, A09, C02, C03	
B. napus	DH	Isolate eH	15 QTLs	A07, C02, A07	[387]
B. nigra	F2	Pathotype 3	*Rcr6*	B03	[107]
B. napus	Association mapping panel (245)	ECD 17/31/31	Two major loci	A02, A03	[248]
B. napus	DH	Pathotype P1	*C03_19.48*	C03	[388]
			C09_58.88	C09	

B. napus ssp. *napobrassica*	*B. napus* ssp. *napobrassica* accessions (124)	2F, 3H, 5I, 6M, 8N, 2B, 3A, 3O, 5C, 5G, 5K, 5L, and 8P	45 SNPs, four PCR based markers	A03, A08	[389]
B. napus	DH	Pathotype P1	*PbBn_di_A02*	A02	[390]
			PbBn_di_A04	A04	
			PbBn_di_C03	C03	
			PbBn_rsp_C03	C03	
B. napus	DH	Field isolate (Lower Silesian Province, Poland)	*Crr3Tsc*	A03	[116]
B. napus	*B. napus* accessions (177)	pathotypes 5X, 3A, 2B, and 3D	13 SNPs	A01, A03, A04, A05, C03, C07 and C09	[391]
B. napus	DH	pathotypes 3A, 3D, 3H	*Rcr10*^{ECD01}	A03	[117]
		pathotypes 3A, 3D, 3H, and 5X	*Rcr9*^{ECD01}	A08	
